# Supplementary material for: Phylogenomic analyses highlight innovation and introgression in the continental radiations of Fagaceae across the Northern Hemisphere
Source: Nat Commun. 2022 Mar 14;13:1320. doi: 10.1038/s41467-022-28917-1 (PMC8921187; doi:10.1038/s41467-022-28917-1)
Supplement: Supplementary file 1 — Supplementary Information [file 41467_2022_28917_MOESM1_ESM.pdf]

Supplementary Information

Phylogenomic analyses highlight innovation and introgression in the continental radiations of Fagaceae across the Northern Hemisphere

Biao-Feng Zhou et al.

Contents

1. Supplementary Figures (Figs. 1 – 16)
2. Supplementary Tables (Tables 1 - 9)

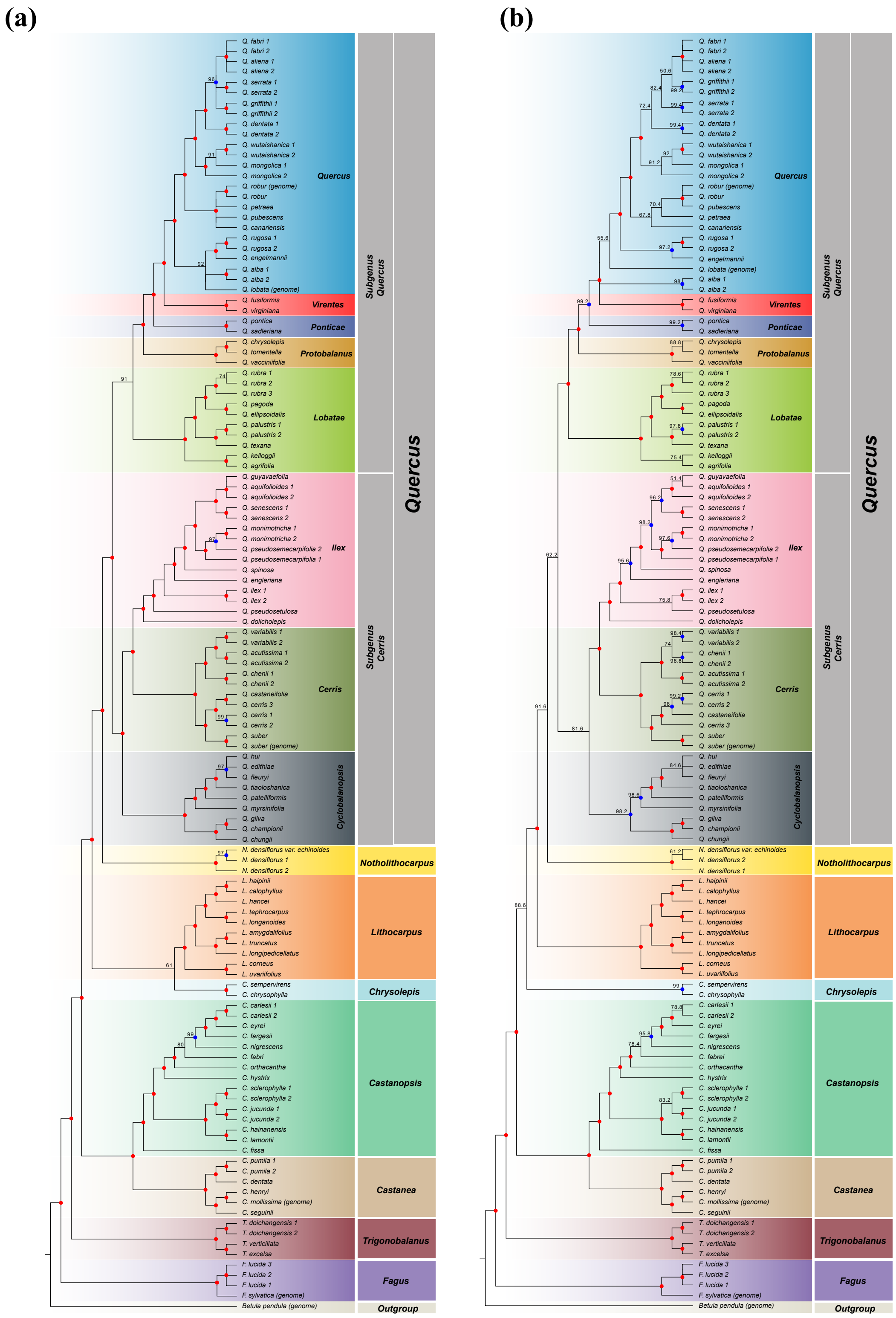

(a)
(b)
Q. fabri 1
Q. fabri 2
Q. aliena 1
Q. aliena 2
Q. serrata 1
Q. serrata 2
Q. griffithii 1
Q. griffithii 2
Q. dentata 1
Q. dentata 2
Q. wutaishanica 1
Q. wutaishanica 2
Q. mongolica 1
Q. mongolica 2
Q. robur (genome)
Q. robur
Q. petraea
Q. pubescens
Q. canariensis
Q. rugosa 1
Q. rugosa 2
Q. engelmannii
Q. alba 1
Q. alba 2
Q. lobata (genome)
Quercus
Q. fusiformis
Q. virginiana
Virentes
Q. pontica
Q. sadleriana
Ponticae
Q. chrysolepis
Q. tomentella
Q. vacciniifolia
Protobalanus
Q. rubra 1
Q. rubra 2
Q. rubra 3
Q. pagoda
Q. ellipsoidalis
Q. palustris 1
Q. palustris 2
Q. texana
Q. kelloggii
Q. agrifolia
Lobatae
Subgenus Quercus
Q. guyavaefolia
Q. aquifolioides 1
Q. aquifolioides 2
Q. senescens 1
Q. senescens 2
Q. monimotricha 1
Q. monimotricha 2
Q. pseudosemecarpifolia 2
Q. pseudosemecarpifolia 1
Q. spinosa
Q. engleriana
Q. ilex 1
Q. ilex 2
Q. pseudosetulosa
Q. dolicholepis
Ilex
Q. variabilis 1
Q. variabilis 2
Q. acutissima 1
Q. acutissima 2
Q. chenii 1
Q. chenii 2
Q. castaneifolia
Q. cerris 3
Q. cerris 1
Q. cerris 2
Q. suber
Q. suber (genome)
Cerris
Q. hui
Q. edithiae
Q. fleuryi
Q. tiaoloshanica
Q. patelliformis
Q. myrsinifolia
Q. gilva
Q. championii
Q. chungii
Cyclobalanopsis
Subgenus Cerris
Quercus
N. densiflorus var. echinoides
N. densiflorus 1
N. densiflorus 2
Notholithocarpus
L. haipinii
L. calophyllus
L. hancei
L. tephrocarpus
L. longanoides
L. amygdalifolius
L. truncatus
L. longipedicellatus
L. corneus
L. uvariifolius
Lithocarpus
C. sempervirens
C. chrysophylla
Chrysolepis
C. carlesii 1
C. carlesii 2
C. eyrei
C. fargesii
C. nigrescens
C. fabri
C. orthacantha
C. hystrix
C. sclerophylla 1
C. sclerophylla 2
C. jucunda 1
C. jucunda 2
C. hainanensis
C. lamontii
C. fissa
Castanopsis
C. pumila 1
C. pumila 2
C. dentata
C. henryi
C. mollissima (genome)
C. seguinii
Castanea
T. doichangensis 1
T. doichangensis 2
T. verticillata
T. excelsa
Trigonobalanus
F. lucida 3
F. lucida 2
F. lucida 1
F. sylvatica (genome)
Fagus
Betula pendula (genome)
Outgroup

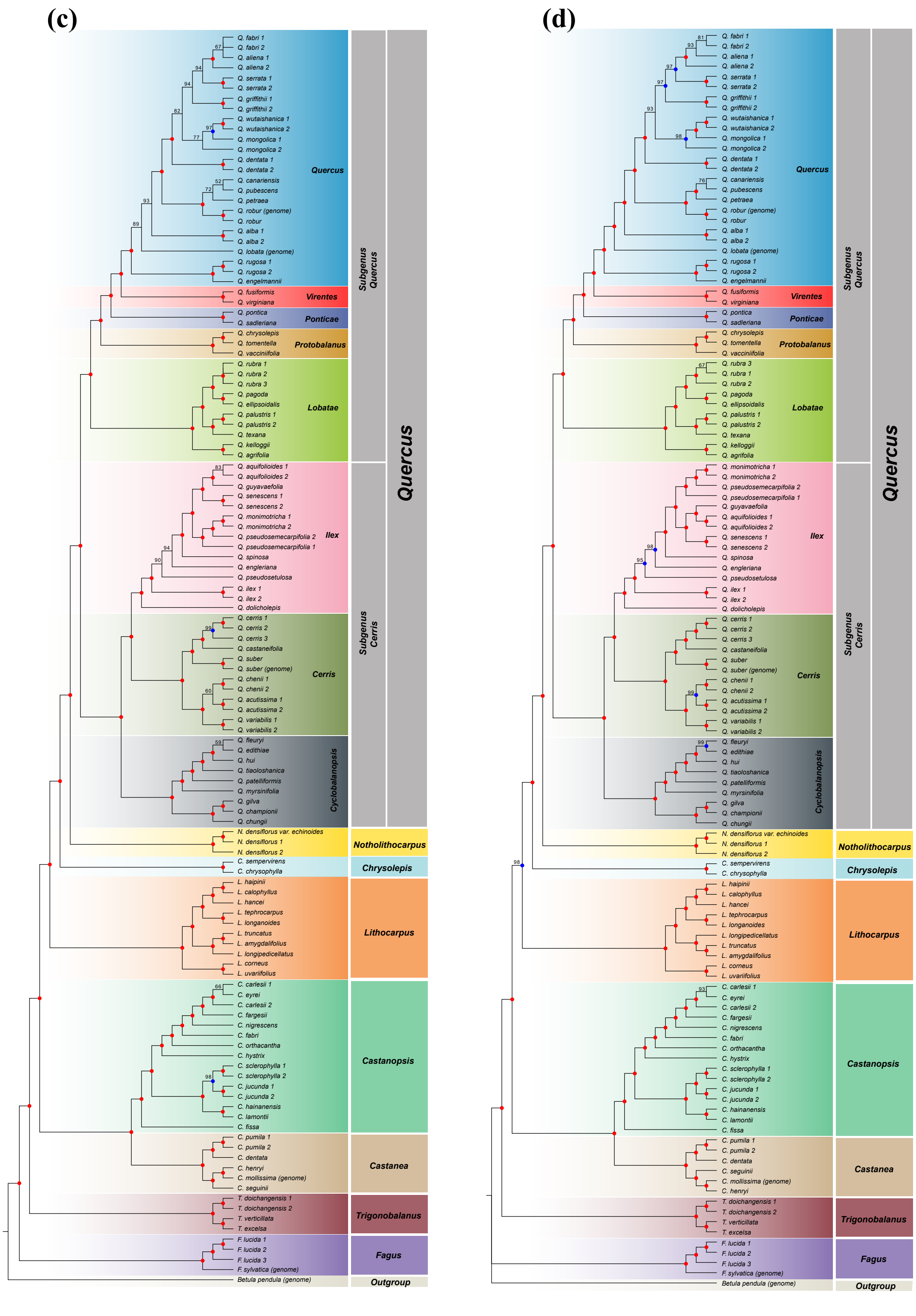
(c)
Q. fabri 1
Q. fabri 2
Q. aliena 1
Q. aliena 2
Q. serrata 1
Q. serrata 2
Q. griffithii 1
Q. griffithii 2
Q. wutaishanica 1
Q. wutaishanica 2
Q. mongolica 1
Q. mongolica 2
Q. dentata 1
Q. dentata 2
Q. canariensis
Q. pubescens
Q. petraea
Q. robur (genome)
Q. robur
Q. alba 1
Q. alba 2
Q. lobata (genome)
Q. rugosa 1
Q. rugosa 2
Q. engelmannii
Quercus
Q. fusiformis
Q. virginiana
Virentes
Q. pontica
Q. sadleriana
Ponticae
Q. chrysolepis
Q. tomentella
Q. vacciniifolia
Protobalanus
Q. rubra 1
Q. rubra 2
Q. rubra 3
Q. pagoda
Q. ellipsoidalis
Q. palustris 1
Q. palustris 2
Q. texana
Q. kelloggii
Q. agrifolia
Lobatae
Subgenus Quercus
Q. aquifolioides 1
Q. aquifolioides 2
Q. guyavaefolia
Q. senescens 1
Q. senescens 2
Q. monimotricha 1
Q. monimotricha 2
Q. pseudosemecarpifolia 2
Q. pseudosemecarpifolia 1
Q. spinosa
Q. engleriana
Q. pseudosetulosa
Q. ilex 1
Q. ilex 2
Q. dolicholepis
Ilex
Q. cerris 1
Q. cerris 2
Q. cerris 3
Q. castaneifolia
Q. suber
Q. suber (genome)
Q. chenii 1
Q. chenii 2
Q. acutissima 1
Q. acutissima 2
Q. variabilis 1
Q. variabilis 2
Cerris
Q. fleuryi
Q. edithiae
Q. hui
Q. tiaoloshanica
Q. patelliformis
Q. myrsinifolia
Q. gilva
Q. championii
Q. chungii
Cyclobalanopsis
Subgenus Cerris
Quercus
N. densiflorus var. echinoides
N. densiflorus 1
N. densiflorus 2
Notholithocarpus
C. sempervirens
C. chrysophylla
Chrysolepis
L. haipinii
L. calophyllus
L. hancei
L. tephrocarpus
L. longanoides
L. truncatus
L. amygdalifolius
L. longipedicellatus
L. corneus
L. uvariifolius
Lithocarpus
C. carlesii 1
C. eyrei
C. carlesii 2
C. fargesii
C. nigrescens
C. fabri
C. orthacantha
C. hystrix
C. sclerophylla 1
C. sclerophylla 2
C. jucunda 1
C. jucunda 2
C. hainanensis
C. lamontii
C. fissa
Castanopsis
C. pumila 1
C. pumila 2
C. dentata
C. henryi
C. mollissima (genome)
C. seguinii
Castanea
T. doichangensis 1
T. doichangensis 2
T. verticillata
T. excelsa
Trigonobalanus
F. lucida 1
F. lucida 2
F. lucida 3
F. sylvatica (genome)
Fagus
Betula pendula (genome)
Outgroup
(d)
Q. fabri 1
Q. fabri 2
Q. aliena 1
Q. aliena 2
Q. serrata 1
Q. serrata 2
Q. griffithii 1
Q. griffithii 2
Q. wutaishanica 1
Q. wutaishanica 2
Q. mongolica 1
Q. mongolica 2
Q. dentata 1
Q. dentata 2
Q. canariensis
Q. pubescens
Q. petraea
Q. robur (genome)
Q. robur
Q. alba 1
Q. alba 2
Q. lobata (genome)
Q. rugosa 1
Q. rugosa 2
Q. engelmannii
Quercus
Q. fusiformis
Q. virginiana
Virentes
Q. pontica
Q. sadleriana
Ponticae
Q. chrysolepis
Q. tomentella
Q. vacciniifolia
Protobalanus
Q. rubra 3
Q. rubra 1
Q. rubra 2
Q. pagoda
Q. ellipsoidalis
Q. palustris 1
Q. palustris 2
Q. texana
Q. kelloggii
Q. agrifolia
Lobatae
Subgenus Quercus
Q. monimotricha 1
Q. monimotricha 2
Q. pseudosemecarpifolia 2
Q. pseudosemecarpifolia 1
Q. guyavaefolia
Q. aquifolioides 1
Q. aquifolioides 2
Q. senescens 1
Q. senescens 2
Q. spinosa
Q. engleriana
Q. pseudosetulosa
Q. ilex 1
Q. ilex 2
Q. dolicholepis
Ilex
Q. cerris 1
Q. cerris 2
Q. cerris 3
Q. castaneifolia
Q. suber
Q. suber (genome)
Q. chenii 1
Q. chenii 2
Q. acutissima 1
Q. acutissima 2
Q. variabilis 1
Q. variabilis 2
Cerris
Q. fleuryi
Q. edithiae
Q. hui
Q. tiaoloshanica
Q. patelliformis
Q. myrsinifolia
Q. gilva
Q. championii
Q. chungii
Cyclobalanopsis
Subgenus Cerris
Quercus
N. densiflorus var. echinoides
N. densiflorus 1
N. densiflorus 2
Notholithocarpus
C. sempervirens
C. chrysophylla
Chrysolepis
L. haipinii
L. calophyllus
L. hancei
L. tephrocarpus
L. longanoides
L. longipedicellatus
L. truncatus
L. amygdalifolius
L. corneus
L. uvariifolius
Lithocarpus
C. carlesii 1
C. eyrei
C. carlesii 2
C. fargesii
C. nigrescens
C. fabri
C. orthacantha
C. hystrix
C. sclerophylla 1
C. sclerophylla 2
C. jucunda 1
C. jucunda 2
C. hainanensis
C. lamontii
C. fissa
Castanopsis
C. pumila 1
C. pumila 2
C. dentata
C. seguinii
C. mollissima (genome)
C. henryi
Castanea
T. doichangensis 1
T. doichangensis 2
T. verticillata
T. excelsa
Trigonobalanus
F. lucida 1
F. lucida 2
F. lucida 3
F. sylvatica (genome)
Fagus
Betula pendula (genome)
Outgroup

Supplementary Figure 1. Phylogenies of Fagaceae inferred using (a) ASTRAL-III, (b) SVDquartets, (c) Maximum Likelihood (ML) and (d) MrBayes based on 2124 nuclear genes. Nodes with phylogenetic supports = 100% are marked with red dots, and nodes with phylogentic support between 95-100% are marked with blue dots. Bootstrap support (BS) for ASTRAL-III, SVDquartets and ML analyses and Bayesian inference (BI) for MrBayes are presented above the branch for each node with BS or BI value < 100%. Branches with BS or BI < 50% are collapsed.

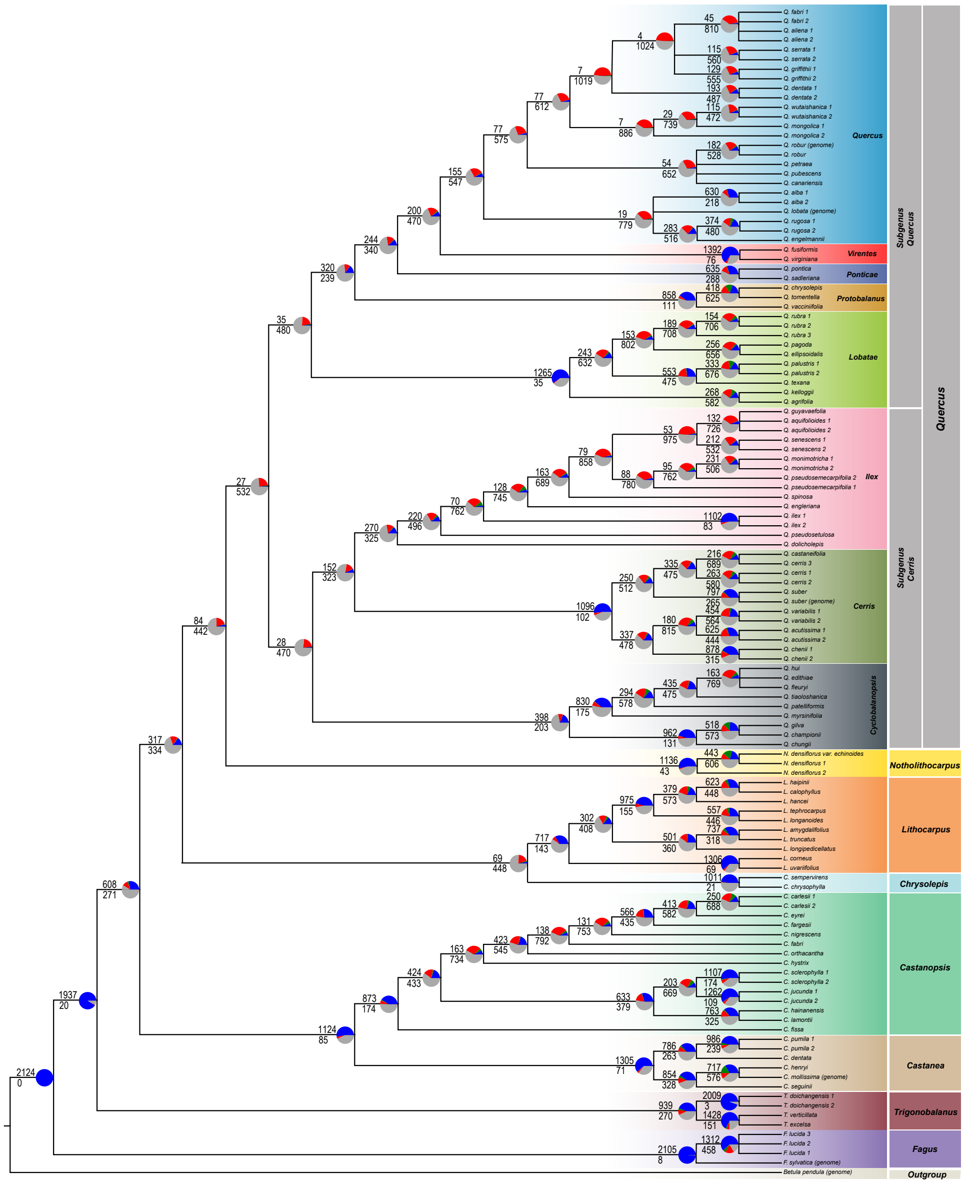

Supplementary Figure 2. Concordance and conflict between nuclear gene trees and species tree demonstrated by PHYPARTS analyses. Coalescent species tree for Fagaceae was inferred using ASTRAL-III based on 2124 nuclear genes, and the same set of 2124 genes were mapped against the species tree. Pie charts next to the nodes indicate the proportions of gene trees supporting the relationship shown (blue), supporting the main alternative bipartition for that clade (green), supporting the remaining alternative bipartitions (red) and uninformative genes (i.e. BS < 50%, grey). The numbers of gene tree concordant and conflicting with a bipartition on the species trees are indicated above and below the branch, respectively.

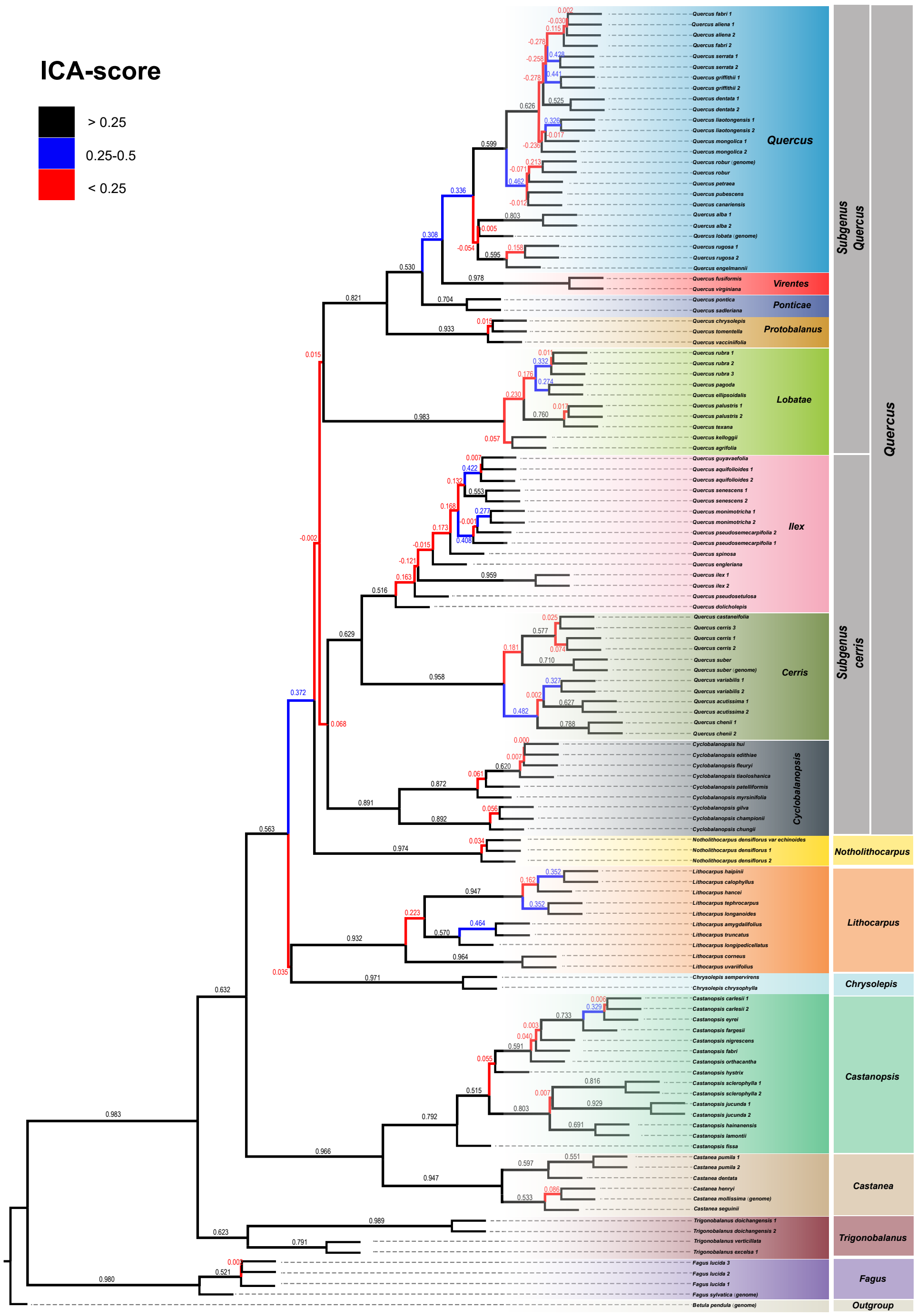

Supplementary Figure 3. Degree of conflict between nuclear gene trees and the species tree on each node. coalescent species tree for Fagaceae was inferred based on 2124 nuclear genes using ASTRAL-III. Number on branches indicate internode certainty (ICA) values estimated from gene trees based on the same 2124 genes. Branches are coloured in black (ICA > 0.5), blue (ICA = 0.25 - 0.5) and red (ICA < 0.25) to show the different level of conflict among gene trees.

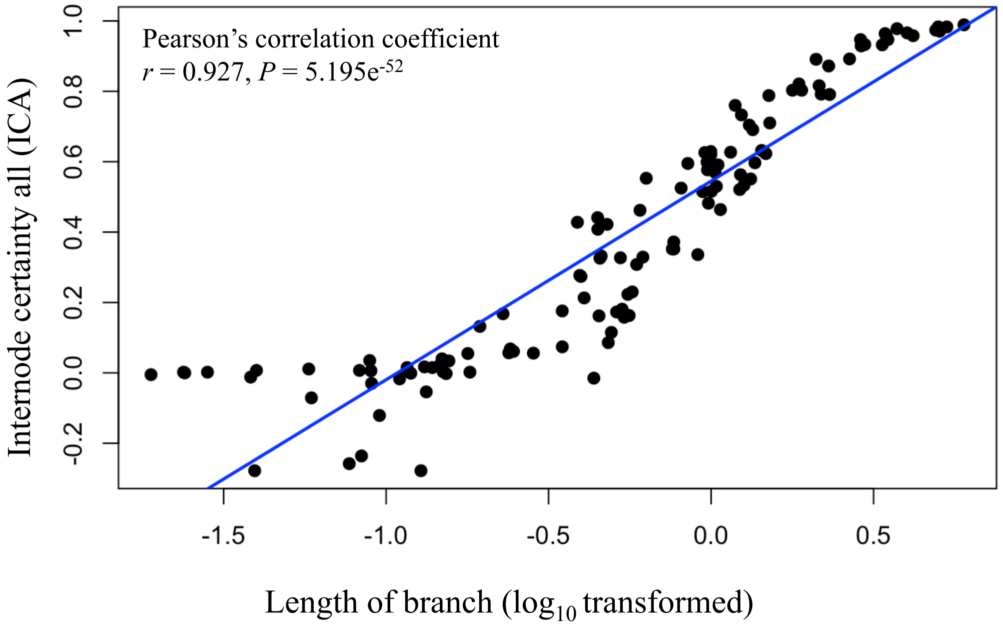

Supplementary Figure 4. The correlation between length of branch and internode certainty (ICA) value. A positive correlation (Pearson's correlation coefficient r = 0.927, P = 5.195e^{-52}; Pearson's correlation test, two-sided) was detected between branch lengths ($\log_{10}$ transformed) and ICA values, consistent with expectation that incomplete lineage sorting (ILS) was responsible for tree conflicts, i.e., the shorter the branch length, the more ILS and conflict among gene trees.

(a)

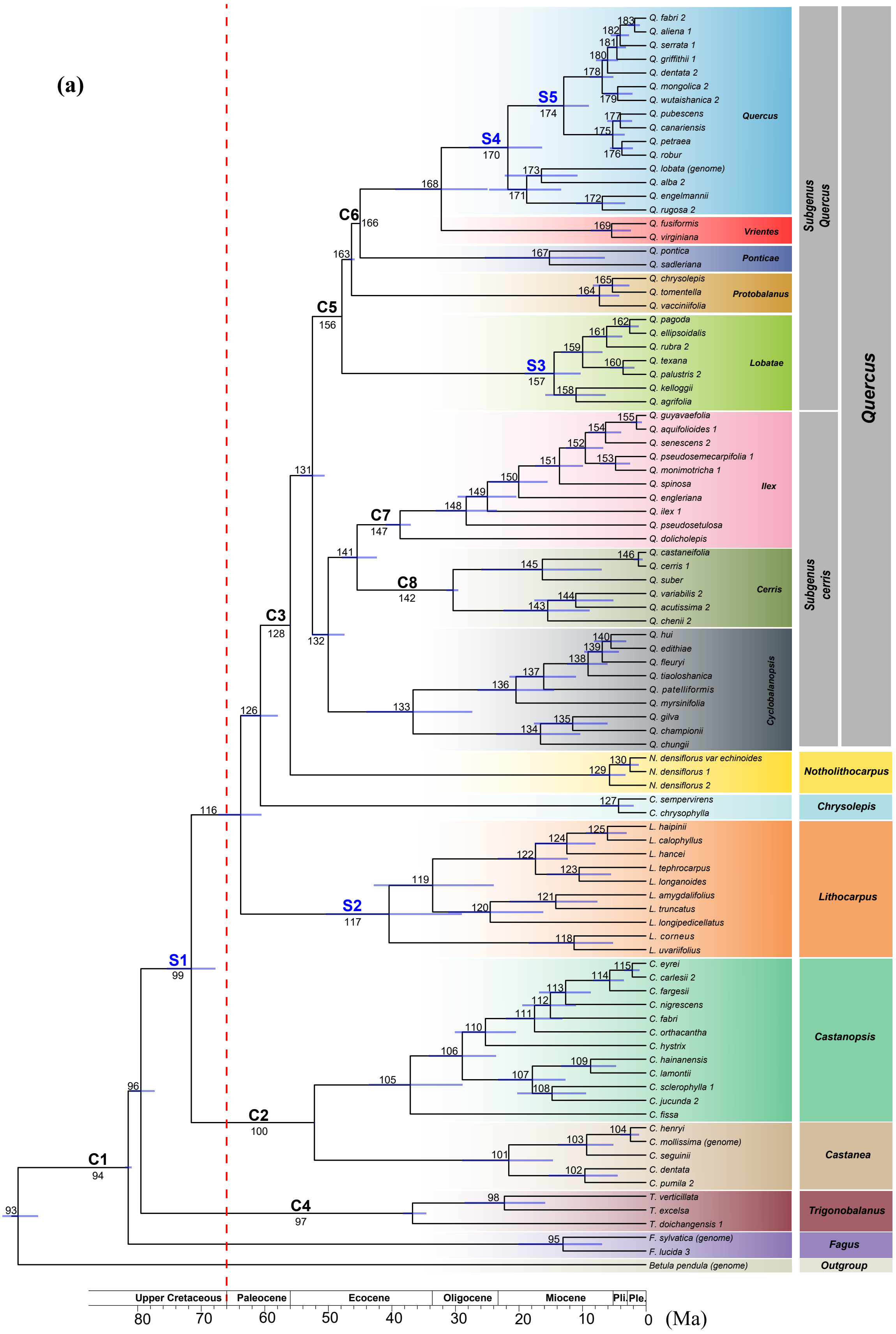
Q. fabri 2
Q. aliena 1
Q. serrata 1
Q. griffithii 1
Q. dentata 2
Q. mongolica 2
Q. wutaishanica 2
Q. pubescens
Q. canariensis
Q. petraea
Q. robur
Q. lobata (genome)
Q. alba 2
Q. engelmannii
Q. rugosa 2
Quercus
Q. fusiformis
Q. virginiana
Virentes
Q. pontica
Q. sadleriana
Ponticae
Q. chrysolepis
Q. tomentella
Q. vacciniifolia
Protobalanus
Q. pagoda
Q. ellipsoidalis
Q. rubra 2
Q. texana
Q. palustris 2
Q. kelloggii
Q. agrifolia
Lobatae
Q. guyavaefolia
Q. aquifolioides 1
Q. senescens 2
Q. pseudosemecarpifolia 1
Q. monimotricha 1
Q. spinosa
Q. engleriana
Q. ilex 1
Q. pseudosetulosa
Q. dolicholepis
Ilex
Q. castaneifolia
Q. cerris 1
Q. suber
Q. variabilis 2
Q. acutissima 2
Q. chenii 2
Cerris
Q. hui
Q. edithiae
Q. fleuryi
Q. tiaoloshanica
Q. patelliformis
Q. myrsinifolia
Q. gilva
Q. championii
Q. chungii
Cyclobalanopsis
N. densiflorus var echinoides
N. densiflorus 1
N. densiflorus 2
C. sempervirens
C. chrysophylla
L. haipinii
L. calophyllus
L. hancei
L. tephrocarpus
L. longanoides
L. amygdalifolius
L. truncatus
L. longipedicellatus
L. corneus
L. uvariifolius
C. eyrei
C. carlesii 2
C. fargesii
C. nigrescens
C. fabri
C. orthacantha
C. hystrix
C. hainanensis
C. lamontii
C. sclerophylla 1
C. jucunda 2
C. fissa
C. henryi
C. mollissima (genome)
C. seguinii
C. dentata
C. pumila 2
T. verticillata
T. excelsa
T. doichangensis 1
F. sylvatica (genome)
F. lucida 3
Betula pendula (genome)
Subgenus Quercus
Subgenus cerris
Quercus
Notholithocarpus
Chrysolepis
Lithocarpus
Castanopsis
Castanea
Trigonobalanus
Fagus
Outgroup
S1
S2
S3
S4
S5
C1
C2
C3
C4
C5
C6
C7
C8
Upper Cretaceous
Paleocene
Ecocene
Oligocene
Miocene
Pli.
Ple.
80
70
60
50
40
30
20
10
0
(Ma)

(b)

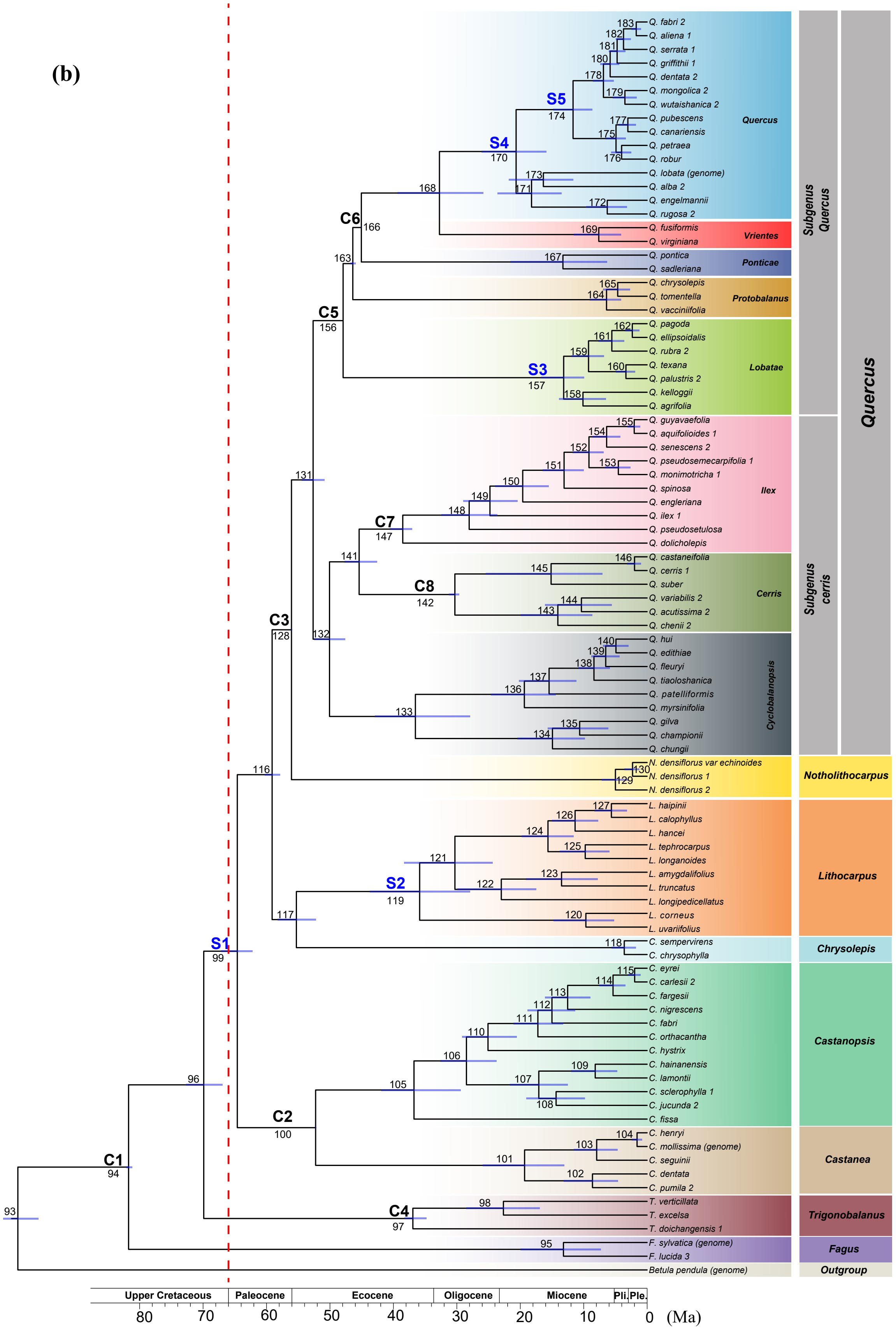
Q. fabri 2
Q. aliena 1
Q. serrata 1
Q. griffithii 1
Q. dentata 2
Q. mongolica 2
Q. wutaishanica 2
Q. pubescens
Q. canariensis
Q. petraea
Q. robur
Q. lobata (genome)
Q. alba 2
Q. engelmannii
Q. rugosa 2
Quercus
Q. fusiformis
Q. virginiana
Virentes
Q. pontica
Q. sadleriana
Ponticae
Q. chrysolepis
Q. tomentella
Q. vacciniifolia
Protobalanus
Q. pagoda
Q. ellipsoidalis
Q. rubra 2
Q. texana
Q. palustris 2
Q. kelloggii
Q. agrifolia
Lobatae
Q. guyavaefolia
Q. aquifolioides 1
Q. senescens 2
Q. pseudosemecarpifolia 1
Q. monimotricha 1
Q. spinosa
Q. engleriana
Q. ilex 1
Q. pseudosetulosa
Q. dolicholepis
Ilex
Q. castaneifolia
Q. cerris 1
Q. suber
Q. variabilis 2
Q. acutissima 2
Q. chenii 2
Cerris
Q. hui
Q. edithiae
Q. fleuryi
Q. tiaoloshanica
Q. patelliformis
Q. myrsinifolia
Q. gilva
Q. championii
Q. chungii
Cyclobalanopsis
Subgenus Quercus
Subgenus cerris
Quercus
N. densiflorus var echinoides
N. densiflorus 1
N. densiflorus 2
Notholithocarpus
L. haipinii
L. calophyllus
L. hancei
L. tephrocarpus
L. longanoides
L. amygdalifolius
L. truncatus
L. longipedicellatus
L. corneus
L. uvariifolius
Lithocarpus
C. sempervirens
C. chrysophylla
Chrysolepis
C. eyrei
C. carlesii 2
C. fargesii
C. nigrescens
C. fabri
C. orthacantha
C. hystrix
C. hainanensis
C. lamontii
C. sclerophylla 1
C. jucunda 2
C. fissa
Castanopsis
C. henryi
C. mollissima (genome)
C. seguinii
C. dentata
C. pumila 2
Castanea
T. verticillata
T. excelsa
T. doichangensis 1
Trigonobalanus
F. sylvatica (genome)
F. lucida 3
Fagus
Betula pendula (genome)
Outgroup
C1
C2
C3
C4
C5
C6
C7
C8
S1
S2
S3
S4
S5
Upper Cretaceous
Paleocene
Eocene
Oligocene
Miocene
Pli.
Ple.
80
70
60
50
40
30
20
10
0
(Ma)

(c)

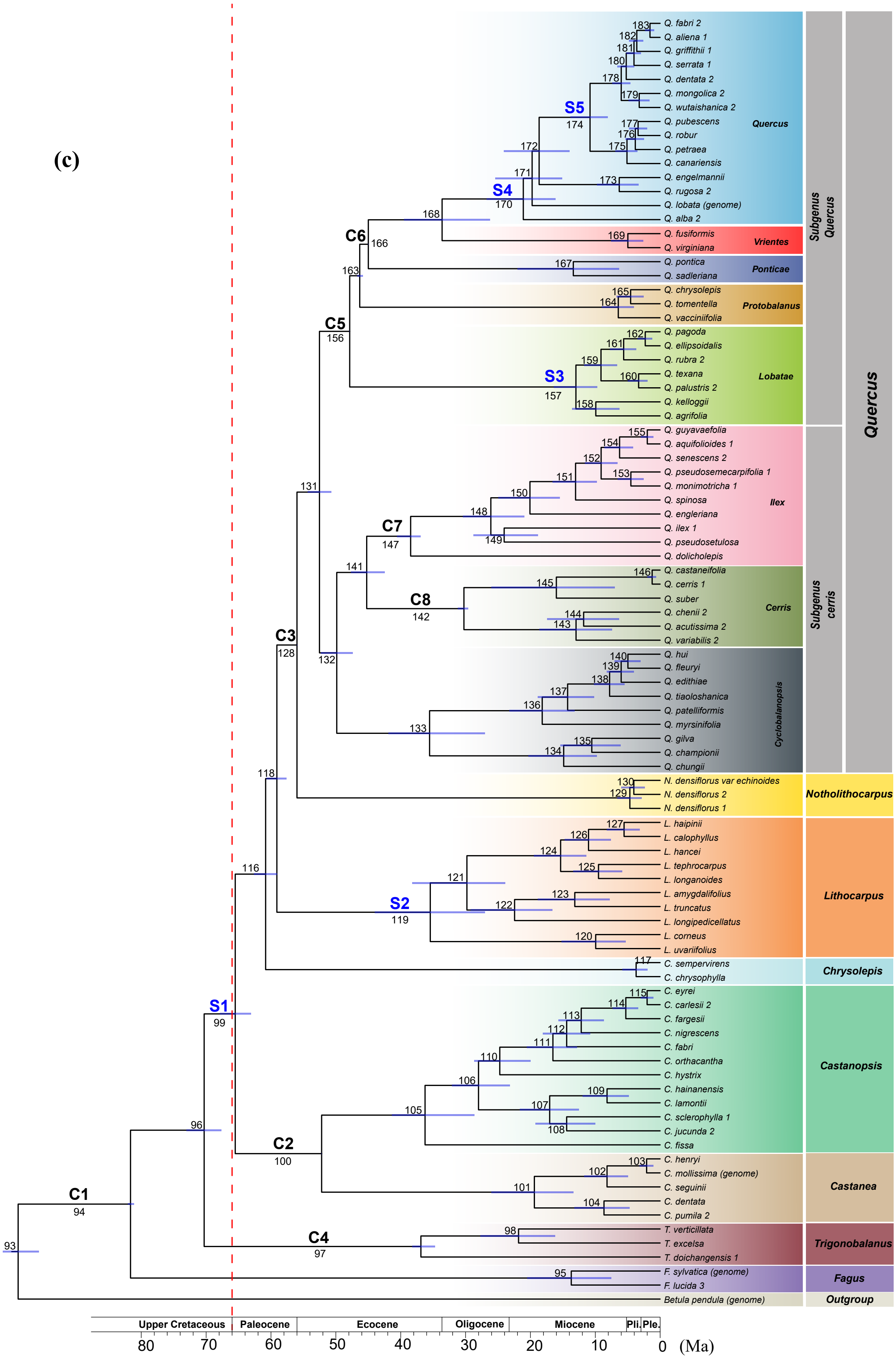

Q. fabri 2
Q. aliena 1
Q. griffithii 1
Q. serrata 1
Q. dentata 2
Q. mongolica 2
Q. wutaishanica 2
Q. pubescens
Q. robur
Q. petraea
Q. canariensis
Q. engelmannii
Q. rugosa 2
Q. lobata (genome)
Q. alba 2
Quercus
Q. fusiformis
Q. virginiana
Virentes
Q. pontica
Q. sadleriana
Ponticae
Q. chrysolepis
Q. tomentella
Q. vacciniifolia
Protobalanus
Q. pagoda
Q. ellipsoidalis
Q. rubra 2
Q. texana
Q. palustris 2
Q. kelloggii
Q. agrifolia
Lobatae
Q. guyavaefolia
Q. aquifolioides 1
Q. senescens 2
Q. pseudosemecarpifolia 1
Q. monimotricha 1
Q. spinosa
Q. engleriana
Q. ilex 1
Q. pseudosetulosa
Q. dolicholepis
Ilex
Q. castaneifolia
Q. cerris 1
Q. suber
Q. chenii 2
Q. acutissima 2
Q. variabilis 2
Cerris
Q. hui
Q. fleuryi
Q. edithiae
Q. tiaoloshanica
Q. patelliformis
Q. myrsinifolia
Q. gilva
Q. championii
Q. chungii
Cyclobalanopsis
Subgenus Quercus
Subgenus cerris
Quercus
N. densiflorus var echinoides
N. densiflorus 2
N. densiflorus 1
Notholithocarpus
L. haipinii
L. calophyllus
L. hancei
L. tephrocarpus
L. longanoides
L. amygdalifolius
L. truncatus
L. longipedicellatus
L. corneus
L. uvariifolius
Lithocarpus
C. sempervirens
C. chrysophylla
Chrysolepis
C. eyrei
C. carlesii 2
C. fargesii
C. nigrescens
C. fabri
C. orthacantha
C. hystrix
C. hainanensis
C. lamontii
C. sclerophylla 1
C. jucunda 2
C. fissa
Castanopsis
C. henryi
C. mollissima (genome)
C. seguinii
C. dentata
C. pumila 2
Castanea
T. verticillata
T. excelsa
T. doichangensis 1
Trigonobalanus
F. sylvatica (genome)
F. lucida 3
Fagus
Betula pendula (genome)
Outgroup
C1
C2
C3
C4
C5
C6
C7
C8
S1
S2
S3
S4
S5
Upper Cretaceous
Paleocene
Eocene
Oligocene
Miocene
Pli.
Ple.
80
70
60
50
40
30
20
10
0
(Ma)

(d)

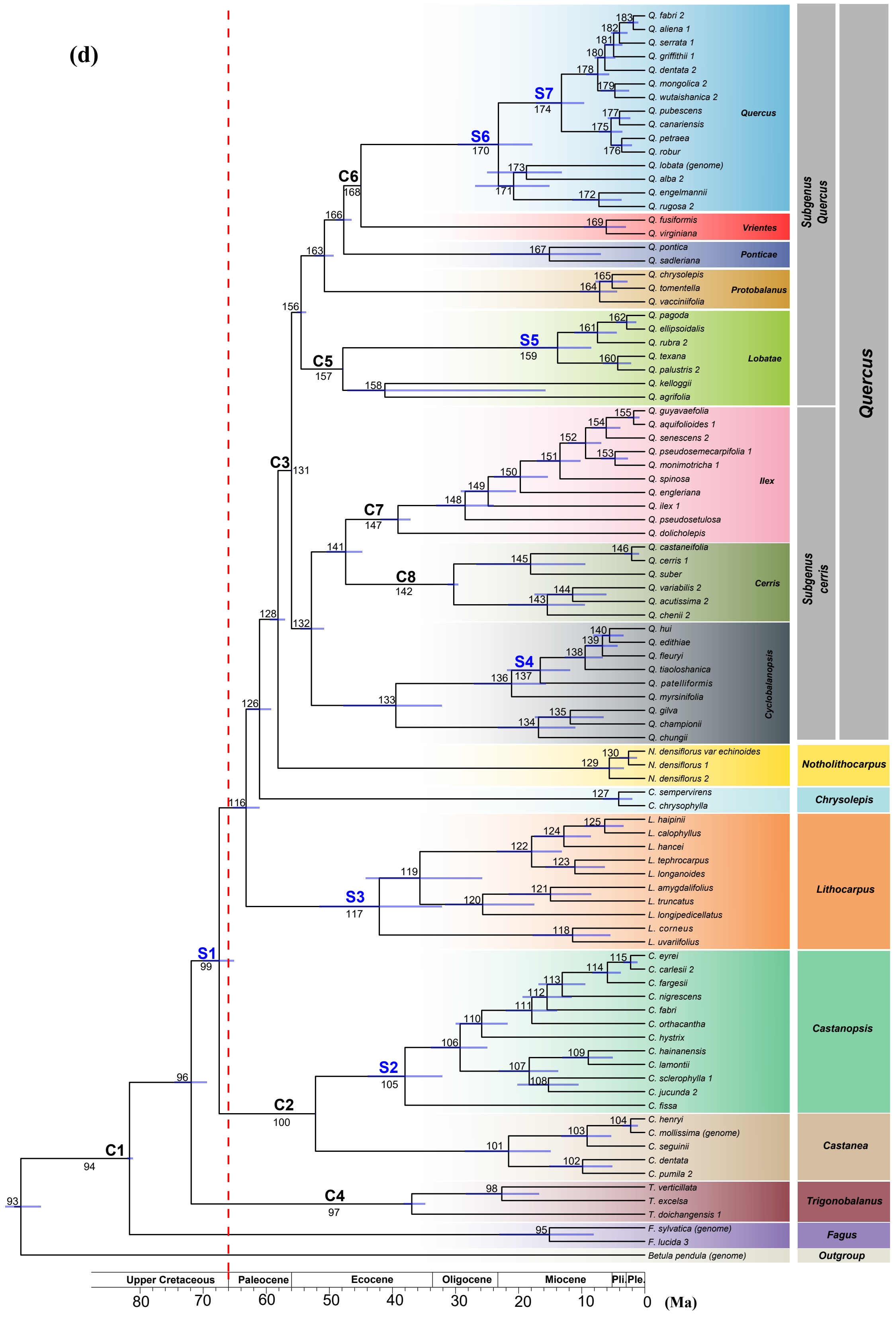

Q. fabri 2
Q. aliena 1
Q. serrata 1
Q. griffithii 1
Q. dentata 2
Q. mongolica 2
Q. wutaishanica 2
Q. pubescens
Q. canariensis
Q. petraea
Q. robur
Q. lobata (genome)
Q. alba 2
Q. engelmannii
Q. rugosa 2
Quercus
Q. fusiformis
Q. virginiana
Vrientes
Q. pontica
Q. sadleriana
Ponticae
Q. chrysolepis
Q. tomentella
Q. vacciniifolia
Protobalanus
Q. pagoda
Q. ellipsoidalis
Q. rubra 2
Q. texana
Q. palustris 2
Q. kelloggii
Q. agrifolia
Lobatae
Q. guyavaefolia
Q. aquifolioides 1
Q. senescens 2
Q. pseudosemecarpifolia 1
Q. monimotricha 1
Q. spinosa
Q. engleriana
Q. ilex 1
Q. pseudosetulosa
Q. dolicholepis
Ilex
Q. castaneifolia
Q. cerris 1
Q. suber
Q. variabilis 2
Q. acutissima 2
Q. chenii 2
Cerris
Q. hui
Q. edithiae
Q. fleuryi
Q. tiaoloshanica
Q. patelliformis
Q. myrsinifolia
Q. gilva
Q. championii
Q. chungii
Cyclobalanopsis
Subgenus Quercus
Subgenus cerris
Quercus
N. densiflorus var echinoides
N. densiflorus 1
N. densiflorus 2
Notholithocarpus
C. sempervirens
C. chrysophylla
Chrysolepis
L. haipinii
L. calophyllus
L. hancei
L. tephrocarpus
L. longanoides
L. amygdalifolius
L. truncatus
L. longipedicellatus
L. corneus
L. uvariifolius
Lithocarpus
C. eyrei
C. carlesii 2
C. fargesii
C. nigrescens
C. fabri
C. orthacantha
C. hystrix
C. hainanensis
C. lamontii
C. sclerophylla 1
C. jucunda 2
C. fissa
Castanopsis
C. henryi
C. mollissima (genome)
C. seguinii
C. dentata
C. pumila 2
Castanea
T. verticillata
T. excelsa
T. doichangensis 1
Trigonobalanus
F. sylvatica (genome)
F. lucida 3
Fagus
Betula pendula (genome)
Outgroup
C1 C2 C3 C4 C5 C6 C7 C8
S1 S2 S3 S4 S5 S6 S7
Upper Cretaceous
Paleocene
Ecocene
Oligocene
Miocene
Pli.
Ple.
80 70 60 50 40 30 20 10 0
(Ma)

(e)

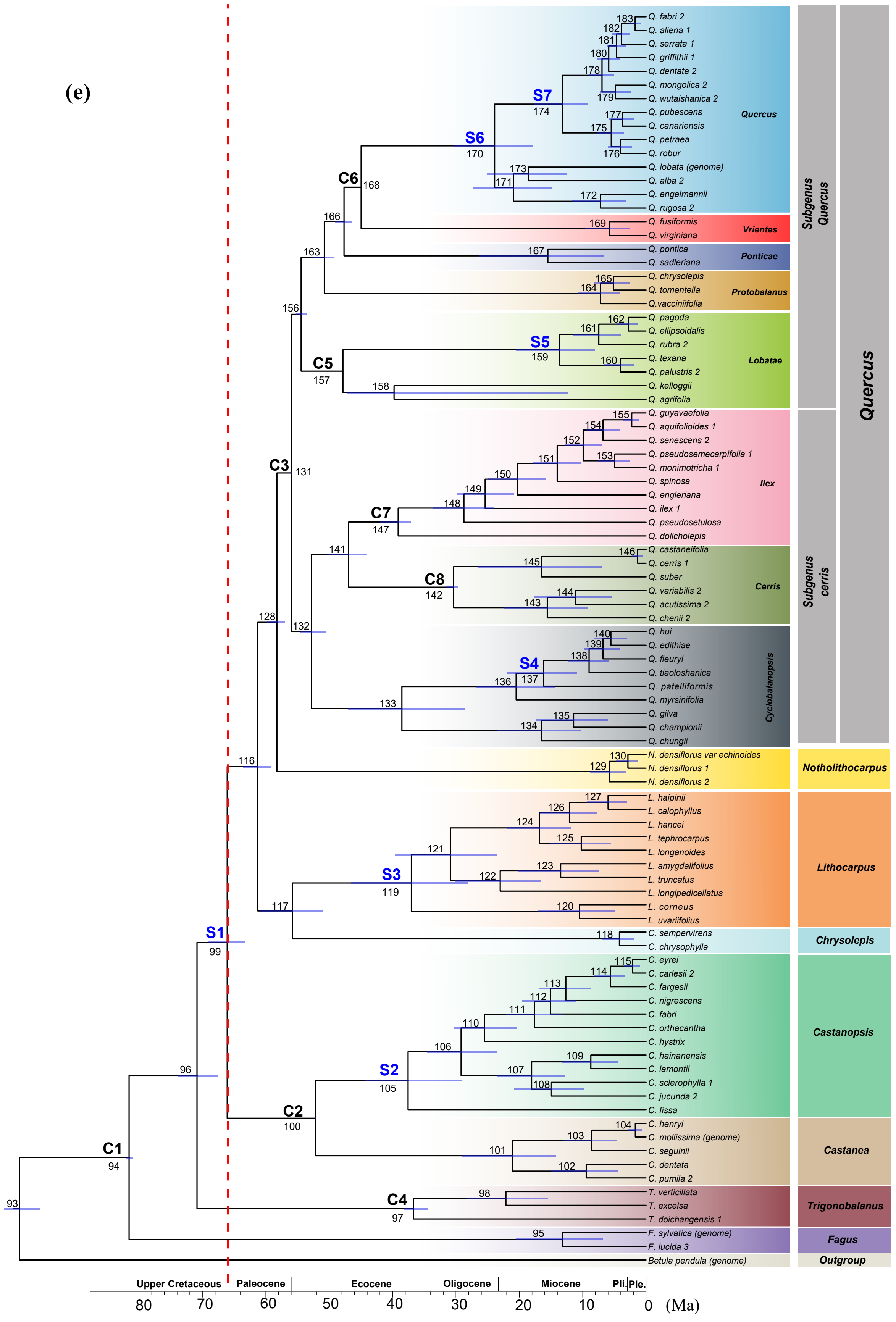

Q. fabri 2
Q. aliena 1
Q. serrata 1
Q. griffithii 1
Q. dentata 2
Q. mongolica 2
Q. wutaishanica 2
Q. pubescens
Q. canariensis
Q. petraea
Q. robur
Q. lobata (genome)
Q. alba 2
Q. engelmannii
Q. rugosa 2
Q. fusiformis
Q. virginiana
Q. pontica
Q. sadleriana
Q. chrysolepis
Q. tomentella
Q.vacciniifolia
Q. pagoda
Q. ellipsoidalis
Q. rubra 2
Q. texana
Q. palustris 2
Q. kelloggii
Q. agrifolia
Q. guyavaefolia
Q. aquifolioides 1
Q. senescens 2
Q. pseudosemecarpifolia 1
Q. monimotricha 1
Q. spinosa
Q. engleriana
Q. ilex 1
Q. pseudosetulosa
Q. dolicholepis
Q. castaneifolia
Q. cerris 1
Q. suber
Q. variabilis 2
Q. acutissima 2
Q. chenii 2
Q. hui
Q. edithiae
Q. fleuryi
Q. tiaoloshanica
Q. patelliformis
Q. myrsinifolia
Q. gilva
Q. championii
Q. chungii
N. densiflorus var echinoides
N. densiflorus 1
N. densiflorus 2
L. haipinii
L. calophyllus
L. hancei
L. tephrocarpus
L. longanoides
L. amygdalifolius
L. truncatus
L. longipedicellatus
L. corneus
L. uvariifolius
C. sempervirens
C. chrysophylla
C. eyrei
C. carlesii 2
C. fargesii
C. nigrescens
C. fabri
C. orthacantha
C. hystrix
C. hainanensis
C. lamontii
C. sclerophylla 1
C. jucunda 2
C. fissa
C. henryi
C. mollissima (genome)
C. seguinii
C. dentata
C. pumila 2
T. verticillata
T. excelsa
T. doichangensis 1
F. sylvatica (genome)
F. lucida 3
Betula pendula (genome)
Quercus
Vrientes
Ponticae
Protobalanus
Lobatae
Ilex
Cerris
Cyclobalanopsis
Notholithocarpus
Lithocarpus
Chrysolepis
Castanopsis
Castanea
Trigonobalanus
Fagus
Outgroup
Subgenus Quercus
Subgenus cerris
Quercus
S1
S2
S3
S4
S5
S6
S7
C1
C2
C3
C4
C5
C6
C7
C8
Upper Cretaceous
Paleocene
Ecocene
Oligocene
Miocene
Pli.
Ple.
80
70
60
50
40
30
20
10
0
(Ma)

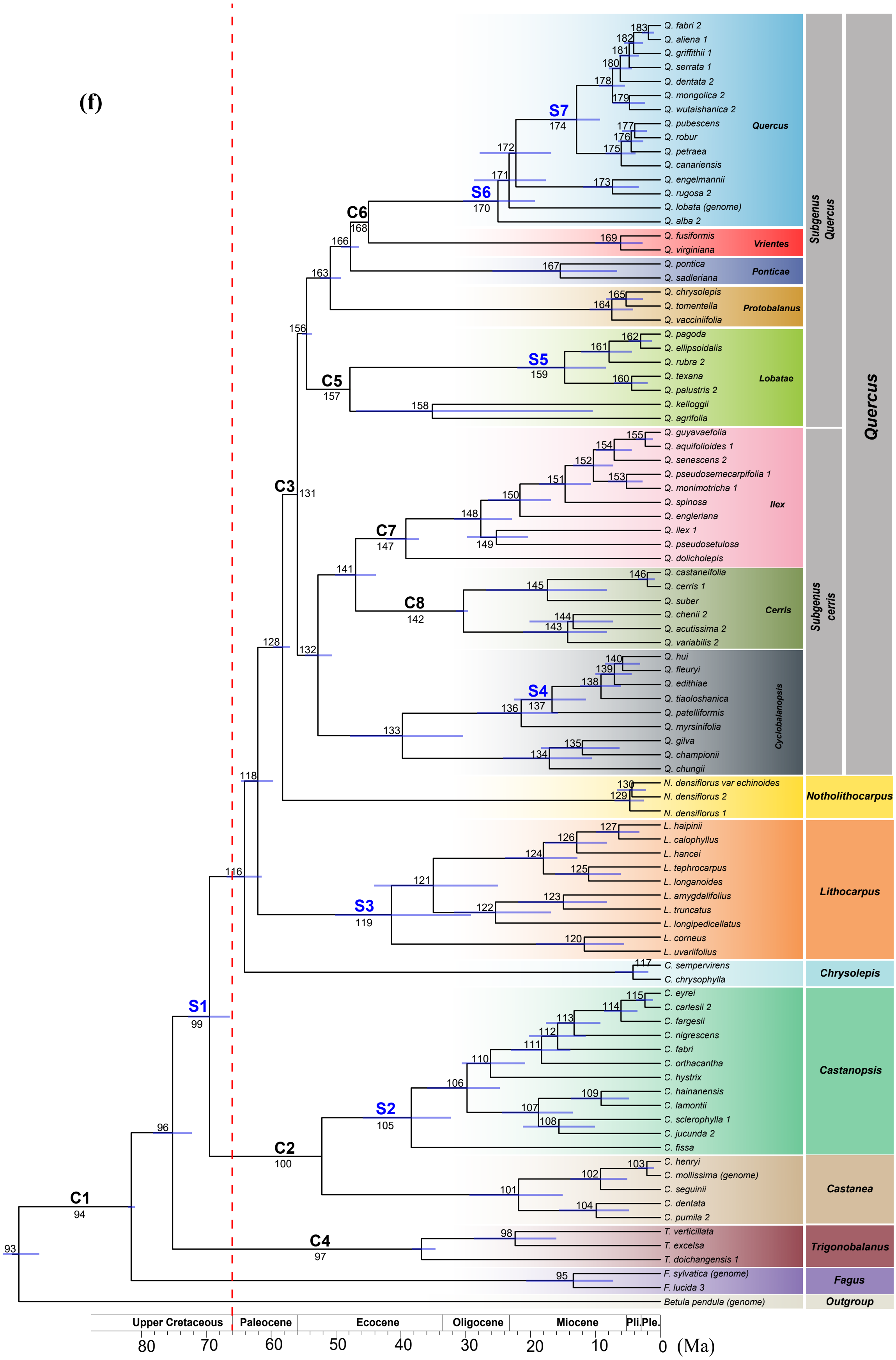

(f)
Q. fabri 2
Q. aliena 1
Q. griffithii 1
Q. serrata 1
Q. dentata 2
Q. mongolica 2
Q. wutaishanica 2
Q. pubescens
Q. robur
Q. petraea
Q. canariensis
Q. engelmannii
Q. rugosa 2
Q. lobata (genome)
Q. alba 2
Quercus
Q. fusiformis
Q. virginiana
Vrientes
Q. pontica
Q. sadleriana
Ponticae
Q. chrysolepis
Q. tomentella
Q. vacciniifolia
Protobalanus
Q. pagoda
Q. ellipsoidalis
Q. rubra 2
Q. texana
Q. palustris 2
Q. kelloggii
Q. agrifolia
Lobatae
Subgenus Quercus
Q. guyavaefolia
Q. aquifolioides 1
Q. senescens 2
Q. pseudosemecarpifolia 1
Q. monimotricha 1
Q. spinosa
Q. engleriana
Q. ilex 1
Q. pseudosetulosa
Q. dolicholepis
Ilex
Q. castaneifolia
Q. cerris 1
Q. suber
Q. chenii 2
Q. acutissima 2
Q. variabilis 2
Cerris
Q. hui
Q. fleuryi
Q. edithiae
Q. tiaoloshanica
Q. patelliformis
Q. myrsinifolia
Q. gilva
Q. championii
Q. chungii
Cyclobalanopsis
Subgenus cerris
Quercus
N. densiflorus var echinoides
N. densiflorus 2
N. densiflorus 1
Notholithocarpus
L. haipinii
L. calophyllus
L. hancei
L. tephrocarpus
L. longanoides
L. amygdalifolius
L. truncatus
L. longipedicellatus
L. corneus
L. uvariifolius
Lithocarpus
C. sempervirens
C. chrysophylla
Chrysolepis
C. eyrei
C. carlesii 2
C. fargesii
C. nigrescens
C. fabri
C. orthacantha
C. hystrix
C. hainanensis
C. lamontii
C. sclerophylla 1
C. jucunda 2
C. fissa
Castanopsis
C. henryi
C. mollissima (genome)
C. seguinii
C. dentata
C. pumila 2
Castanea
T. verticillata
T. excelsa
T. doichangensis 1
Trigonobalanus
F. sylvatica (genome)
F. lucida 3
Fagus
Betula pendula (genome)
Outgroup
C1 C2 C3 C4 C5 C6 C7 C8
S1 S2 S3 S4 S5 S6 S7
Upper Cretaceous
Paleocene
Ecocene
Oligocene
Miocene
Pli.
Ple.
80 70 60 50 40 30 20 10 0 (Ma)

(g)

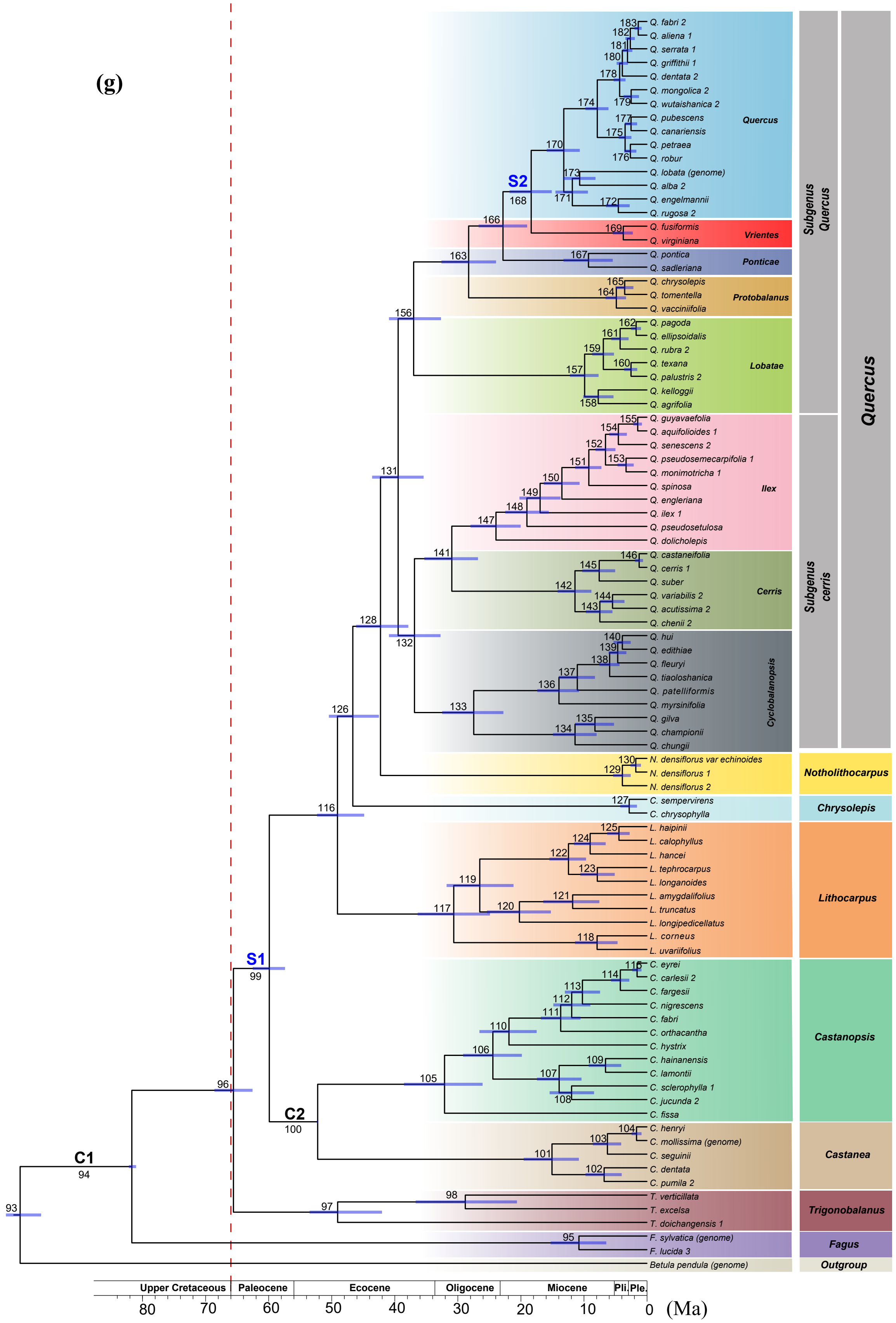

S2
S1
C1
C2
Q. fabri 2
Q. aliena 1
Q. serrata 1
Q. griffithii 1
Q. dentata 2
Q. mongolica 2
Q. wutaishanica 2
Q. pubescens
Q. canariensis
Q. petraea
Q. robur
Q. lobata (genome)
Q. alba 2
Q. engelmannii
Q. rugosa 2
Q. fusiformis
Q. virginiana
Q. pontica
Q. sadleriana
Q. chrysolepis
Q. tomentella
Q. vacciniifolia
Q. pagoda
Q. ellipsoidalis
Q. rubra 2
Q. texana
Q. palustris 2
Q. kelloggii
Q. agrifolia
Q. guyavaefolia
Q. aquifolioides 1
Q. senescens 2
Q. pseudosemecarpifolia 1
Q. monimotricha 1
Q. spinosa
Q. engleriana
Q. ilex 1
Q. pseudosetulosa
Q. dolicholepis
Q. castaneifolia
Q. cerris 1
Q. suber
Q. variabilis 2
Q. acutissima 2
Q. chenii 2
Q. hui
Q. edithiae
Q. fleuryi
Q. tiaoloshanica
Q. patelliformis
Q. myrsinifolia
Q. gilva
Q. championii
Q. chungii
N. densiflorus var echinoides
N. densiflorus 1
N. densiflorus 2
C. sempervirens
C. chrysophylla
L. haipinii
L. calophyllus
L. hancei
L. tephrocarpus
L. longanoides
L. amygdalifolius
L. truncatus
L. longipedicellatus
L. corneus
L. uvariifolius
C. eyrei
C. carlesii 2
C. fargesii
C. nigrescens
C. fabri
C. orthacantha
C. hystrix
C. hainanensis
C. lamontii
C. sclerophylla 1
C. jucunda 2
C. fissa
C. henryi
C. mollissima (genome)
C. seguinii
C. dentata
C. pumila 2
T. verticillata
T. excelsa
T. doichangensis 1
F. sylvatica (genome)
F. lucida 3
Betula pendula (genome)
Quercus
Vrientes
Ponticae
Protobalanus
Lobatae
Ilex
Cerris
Cyclobalanopsis
Subgenus Quercus
Subgenus cerris
Quercus
Notholithocarpus
Chrysolepis
Lithocarpus
Castanopsis
Castanea
Trigonobalanus
Fagus
Outgroup
Upper Cretaceous
Paleocene
Eocene
Oligocene
Miocene
Pli.
Ple.
80
70
60
50
40
30
20
10
0
(Ma)

(h)

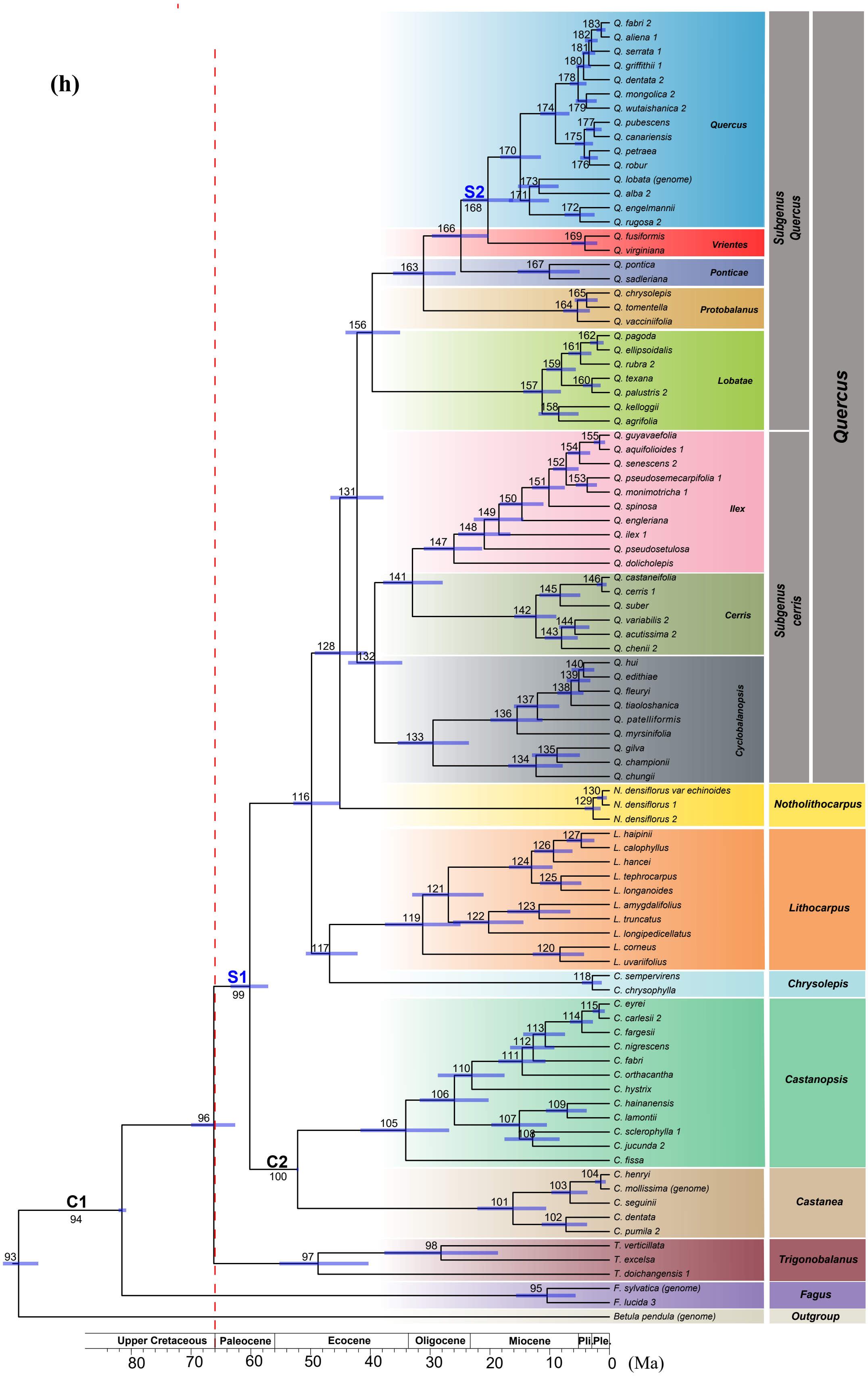

Q. fabri 2
Q. aliena 1
Q. serrata 1
Q. griffithii 1
Q. dentata 2
Q. mongolica 2
Q. wutaishanica 2
Q. pubescens
Q. canariensis
Q. petraea
Q. robur
Q. lobata (genome)
Q. alba 2
Q. engelmannii
Q. rugosa 2
Quercus
Q. fusiformis
Q. virginiana
Vrientes
Q. pontica
Q. sadleriana
Ponticae
Q. chrysolepis
Q. tomentella
Q. vacciniifolia
Protobalanus
Q. pagoda
Q. ellipsoidalis
Q. rubra 2
Q. texana
Q. palustris 2
Q. kelloggii
Q. agrifolia
Lobatae
Subgenus Quercus
Q. guyavaefolia
Q. aquifolioides 1
Q. senescens 2
Q. pseudosemecarpifolia 1
Q. monimotricha 1
Q. spinosa
Q. engleriana
Q. ilex 1
Q. pseudosetulosa
Q. dolicholepis
Ilex
Q. castaneifolia
Q. cerris 1
Q. suber
Q. variabilis 2
Q. acutissima 2
Q. chenii 2
Cerris
Q. hui
Q. edithiae
Q. fleuryi
Q. tiaoloshanica
Q. patelliformis
Q. myrsinifolia
Q. gilva
Q. championii
Q. chungii
Cyclobalanopsis
Subgenus cerris
Quercus
N. densiflorus var echinoides
N. densiflorus 1
N. densiflorus 2
Notholithocarpus
L. haipinii
L. calophyllus
L. hancei
L. tephrocarpus
L. longanoides
L. amygdalifolius
L. truncatus
L. longipedicellatus
L. corneus
L. uvariifolius
Lithocarpus
C. sempervirens
C. chrysophylla
Chrysolepis
C. eyrei
C. carlesii 2
C. fargesii
C. nigrescens
C. fabri
C. orthacantha
C. hystrix
C. hainanensis
C. lamontii
C. sclerophylla 1
C. jucunda 2
C. fissa
Castanopsis
C. henryi
C. mollissima (genome)
C. seguinii
C. dentata
C. pumila 2
Castanea
T. verticillata
T. excelsa
T. doichangensis 1
Trigonobalanus
F. sylvatica (genome)
F. lucida 3
Fagus
Betula pendula (genome)
Outgroup
S2
168
S1
99
C2
100
C1
94
Upper Cretaceous
Paleocene
Ecocene
Oligocene
Miocene
Pli.
Ple.
80
70
60
50
40
30
20
10
0
(Ma)

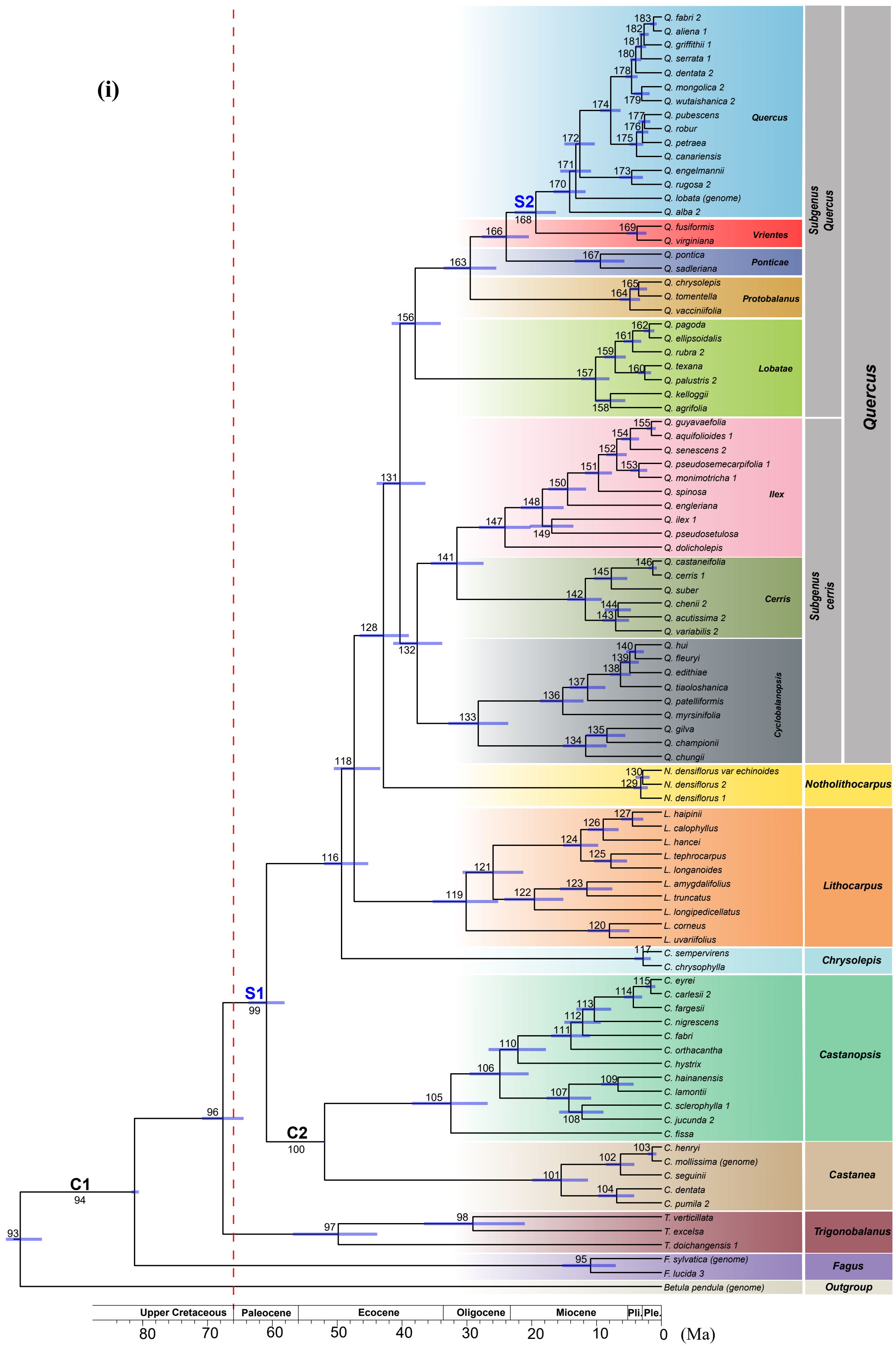

(i)
Q. fabri 2
Q. aliena 1
Q. griffithii 1
Q. serrata 1
Q. dentata 2
Q. mongolica 2
Q. wutaishanica 2
Q. pubescens
Q. robur
Q. petraea
Q. canariensis
Q. engelmannii
Q. rugosa 2
Q. lobata (genome)
Q. alba 2
Quercus
S2
Q. fusiformis
Q. virginiana
Vrientes
Q. pontica
Q. sadleriana
Ponticae
Q. chrysolepis
Q. tomentella
Q. vacciniifolia
Protobalanus
Q. pagoda
Q. ellipsoidalis
Q. rubra 2
Q. texana
Q. palustris 2
Q. kelloggii
Q. agrifolia
Lobatae
Subgenus Quercus
Q. guyavaefolia
Q. aquifolioides 1
Q. senescens 2
Q. pseudosemecarpifolia 1
Q. monimotricha 1
Q. spinosa
Q. engleriana
Q. ilex 1
Q. pseudosetulosa
Q. dolicholepis
Ilex
Q. castaneifolia
Q. cerris 1
Q. suber
Q. chenii 2
Q. acutissima 2
Q. variabilis 2
Cerris
Q. hui
Q. fleuryi
Q. edithiae
Q. tiaoloshanica
Q. patelliformis
Q. myrsinifolia
Q. gilva
Q. championii
Q. chungii
Cyclobalanopsis
Subgenus cerris
Quercus
N. densiflorus var echinoides
N. densiflorus 2
N. densiflorus 1
Notholithocarpus
L. haipinii
L. calophyllus
L. hancei
L. tephrocarpus
L. longanoides
L. amygdalifolius
L. truncatus
L. longipedicellatus
L. corneus
L. uvariifolius
Lithocarpus
C. sempervirens
C. chrysophylla
Chrysolepis
S1
C. eyrei
C. carlesii 2
C. fargesii
C. nigrescens
C. fabri
C. orthacantha
C. hystrix
C. hainanensis
C. lamontii
C. sclerophylla 1
C. jucunda 2
C. fissa
Castanopsis
C2
C. henryi
C. mollissima (genome)
C. seguinii
C. dentata
C. pumila 2
Castanea
C1
T. verticillata
T. excelsa
T. doichangensis 1
Trigonobalanus
F. sylvatica (genome)
F. lucida 3
Fagus
Betula pendula (genome)
Outgroup
Upper Cretaceous
Paleocene
Ecocene
Oligocene
Miocene
Pli.
Ple.
80
70
60
50
40
30
20
10
0
(Ma)

Supplementary Figure 5. Estimated divergence time of Fagaceae based on nuclear data. Chronogram derived from Maximum Likelihood tree without constraints (a, d and g), constrained to ASTRAL-III topology (b, e and h), or constrained to SVDquartets topology (c, f and i). Light blue bars on nodes represent 95% confidence intervals of divergence time estimates. The dashed vertical red line represents the age of the Cretaceous-Paleogene boundary (66 Ma). In a-f, fossil calibration nodes are indicated with C1 – C8 (stem calibration nodes in a-c, crown calibration nodes in d-f). In g-i, fossil calibration nodes are indicated with C1 – C2. S1- S5 in a-c, S1-S7 in e-f and S1-S2 in g-i indicate nodes with a shift in diversification rate revealed by BAMM and/or MEDUSA. The geological timescale is shown at the bottom. Ma, million years ago; Pli, Pliocene; Ple, Pleistocene.

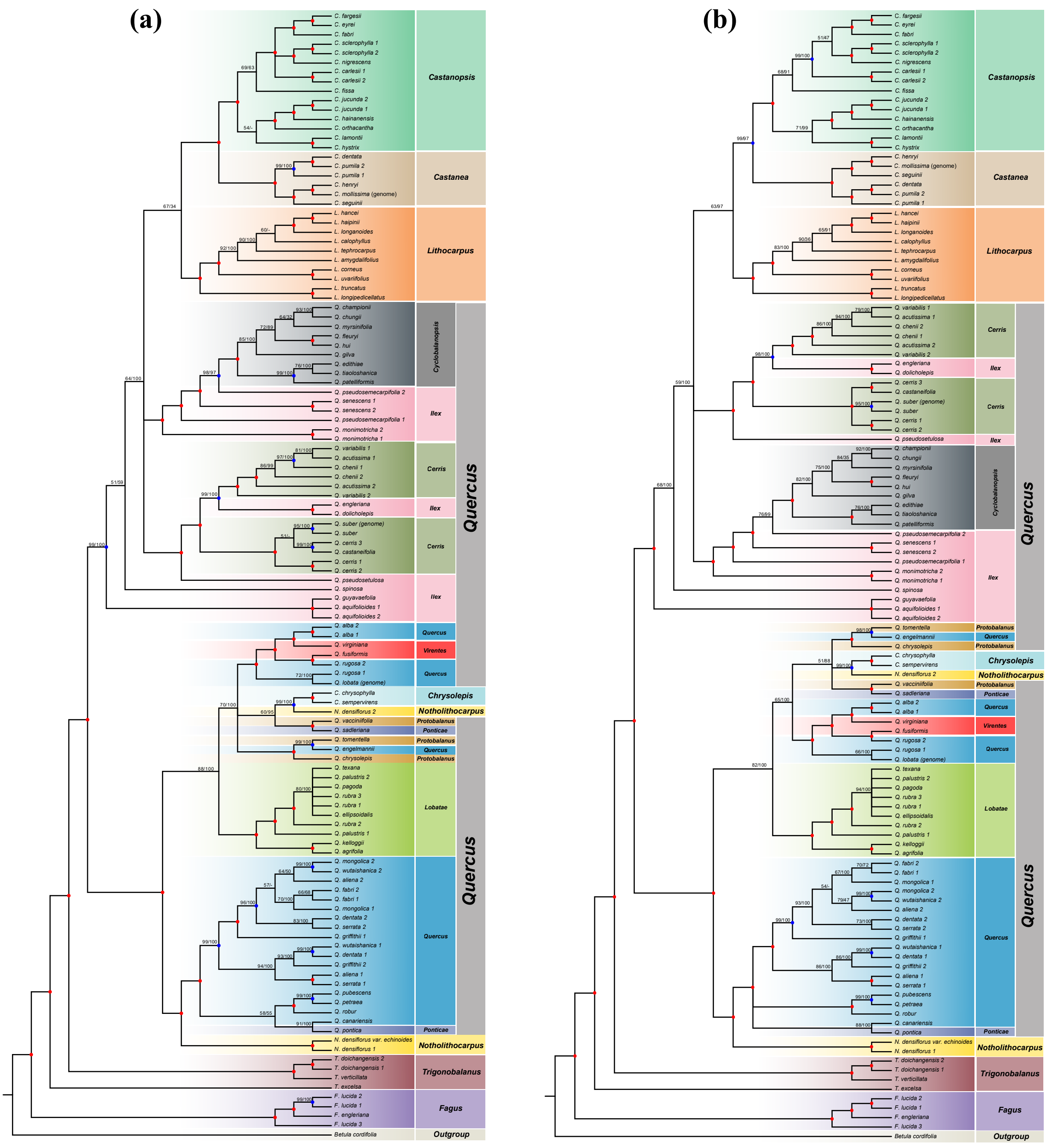
(a)
(b)
Castanopsis
Castanea
Lithocarpus
Quercus
Chrysolepis
Notholithocarpus
Trigonobalanus
Fagus
Outgroup

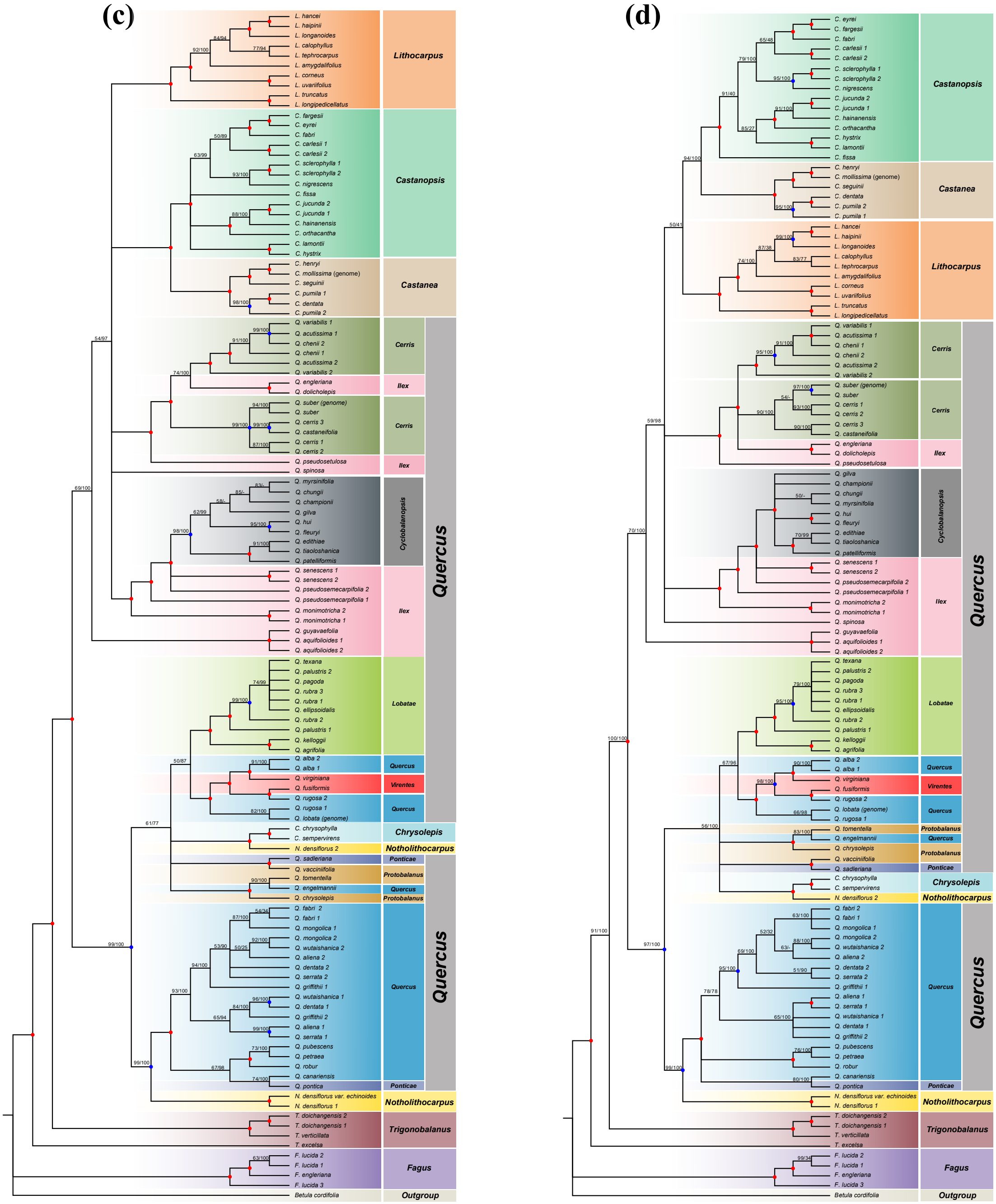

Supplementary Figure 6. ML phylogenies of Fagaceae inferred from 76 plastid genes using different partitioning schemes and alignments. (a) Full nucleotide sequences partitioned by both gene and codon position, (b) un-partitioned full nucleotide sequence data, (c) partitioned amino acid sequence data, and (d) partitioned nucleotide sequence data without 3rd codons. Nodes with phylogenetic support = 100% in both ML and Bayesian analyses are marked with red dots, and nodes with support between 95%-99% in both ML and Bayesian analyses are marked with blue dots. Bootstrap support (BS) for ML analyses and Bayesian inference (BI) for MrBayes are presented above branch for each node with BS or BI value < 100%. Branches with BS < 50% are collapsed.

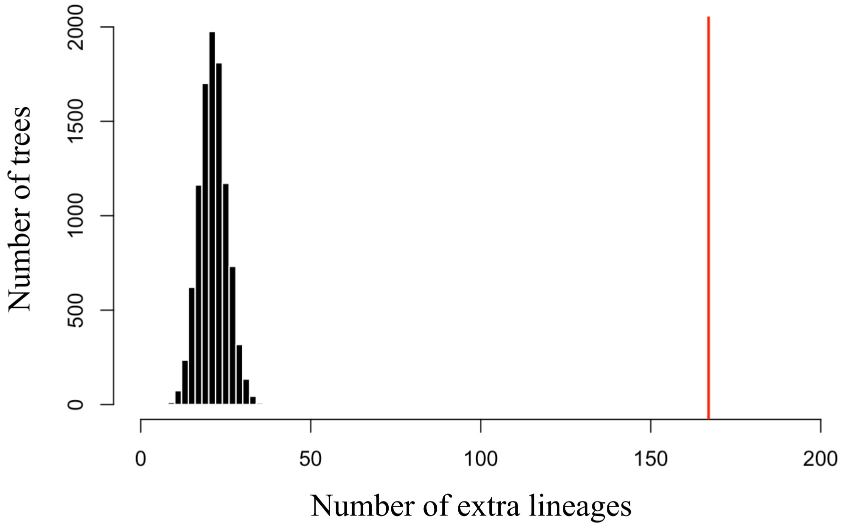

Supplementary Figure 7. The number of extra lineages required to reconcile gene trees simulated under a strict coalescent model. Black bars show the distribution of simulated plastome trees. The vertical red line represents the number of extra lineages observed in plastid gene trees.

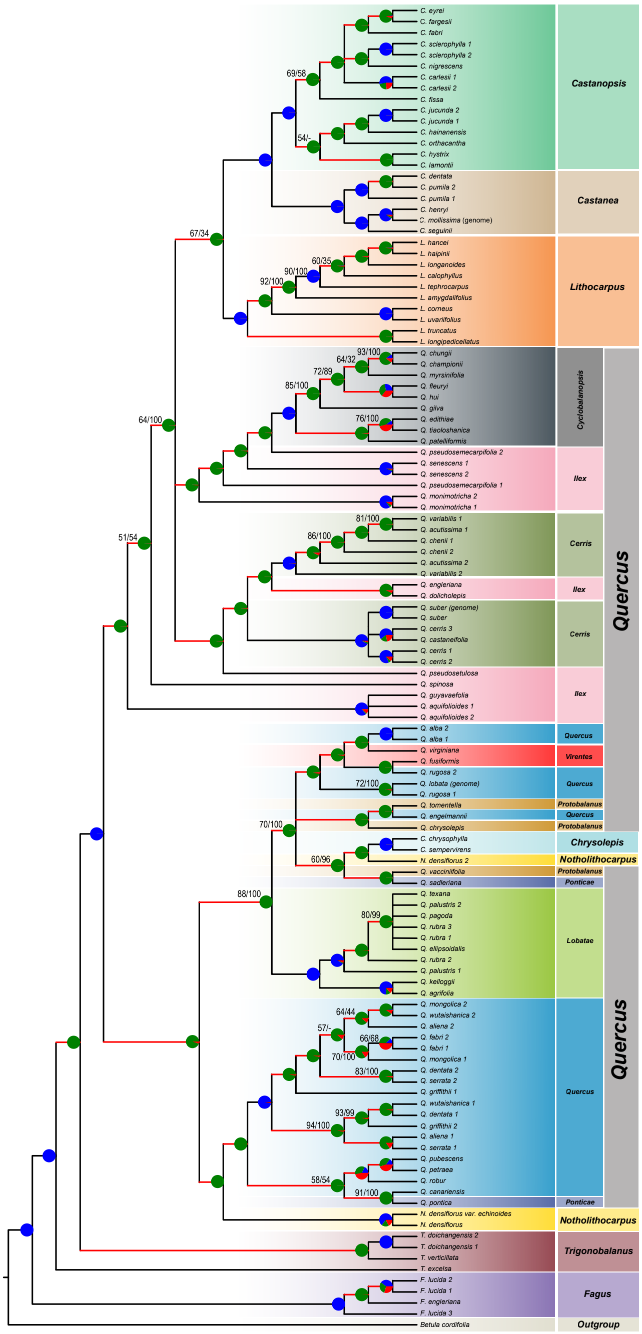

Supplementary Figure 8. Conflict between plastome tree and simulated plastid gene trees. The plastome tree of Fagaceae was inferred from 76 plastid genes using Maximum Likelihood (ML) analyses. Branches are colored in red for bipartitions that are discordant between plastome tree and ASTRAL-III nuclear species tree. Branches with support < 50% on ML analyses were collapsed. Bootstrap (BS) values for ML analyses and Bayesian inference (BI) by MrBayes are presented for nodes with BS < 95% or BI < 95%. Nodes with conflicting bipartitions revealed by ML and MrBayes are indicated with hyphens (-). Pie charts indicate the proportions of 10,000 simulated plastid gene trees supporting the relationship shown (blue), supporting the main alternative bipartition for that clade (green), supporting the remaining alternative bipartitions (red).

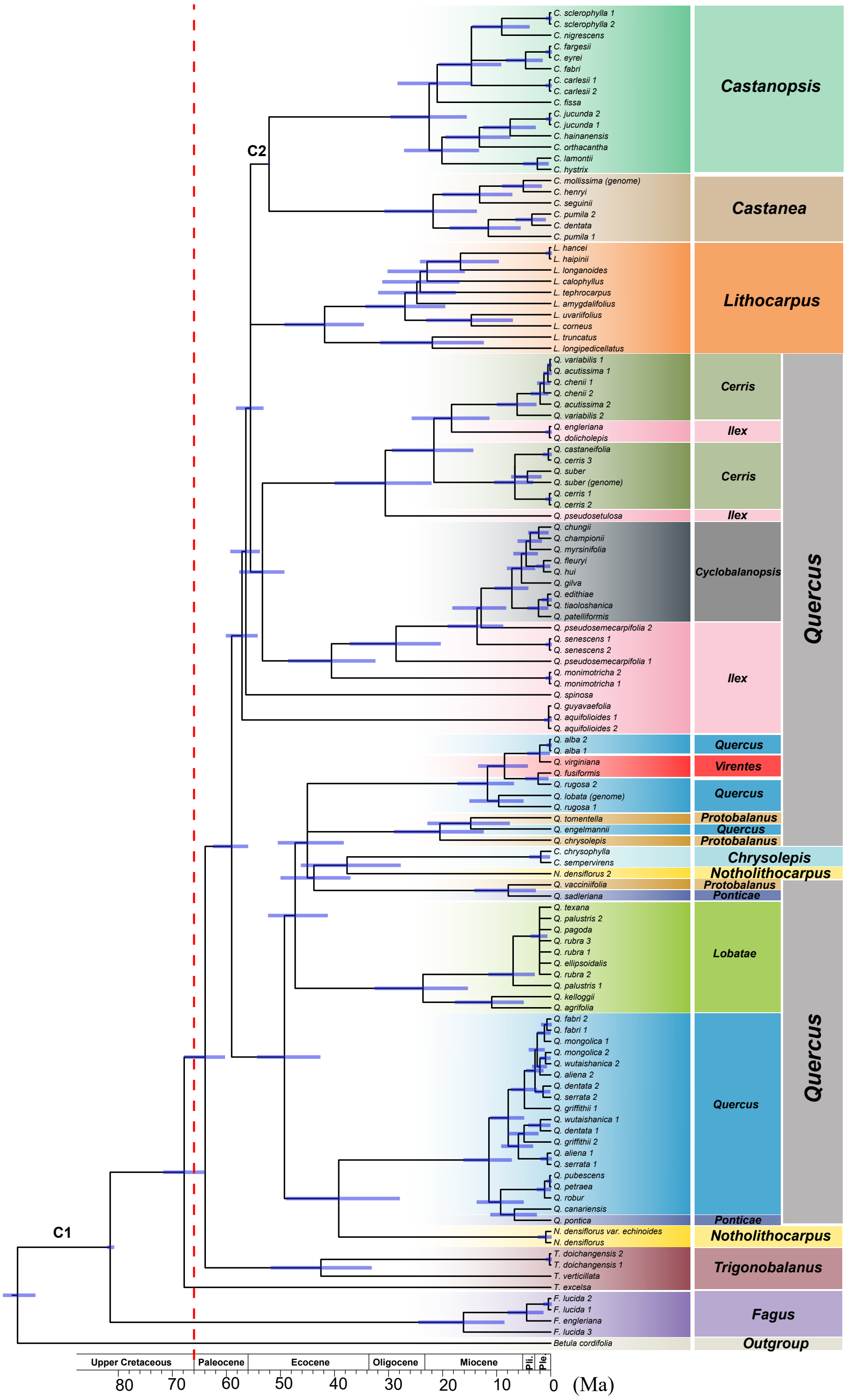

Supplementary Figure 9. Chronogram derived from the Maximum Likelihood tree based on 76 plastid genes. Light blue bars on nodes represent 95% confidence intervals of divergence time estimates. The dashed vertical red line represents the age of the Cretaceous-Paleogene boundary (66 MYA). The geological timescale at the bottom is in million years (Ma). C1 and C2 are two fossil calibration points.

(a)

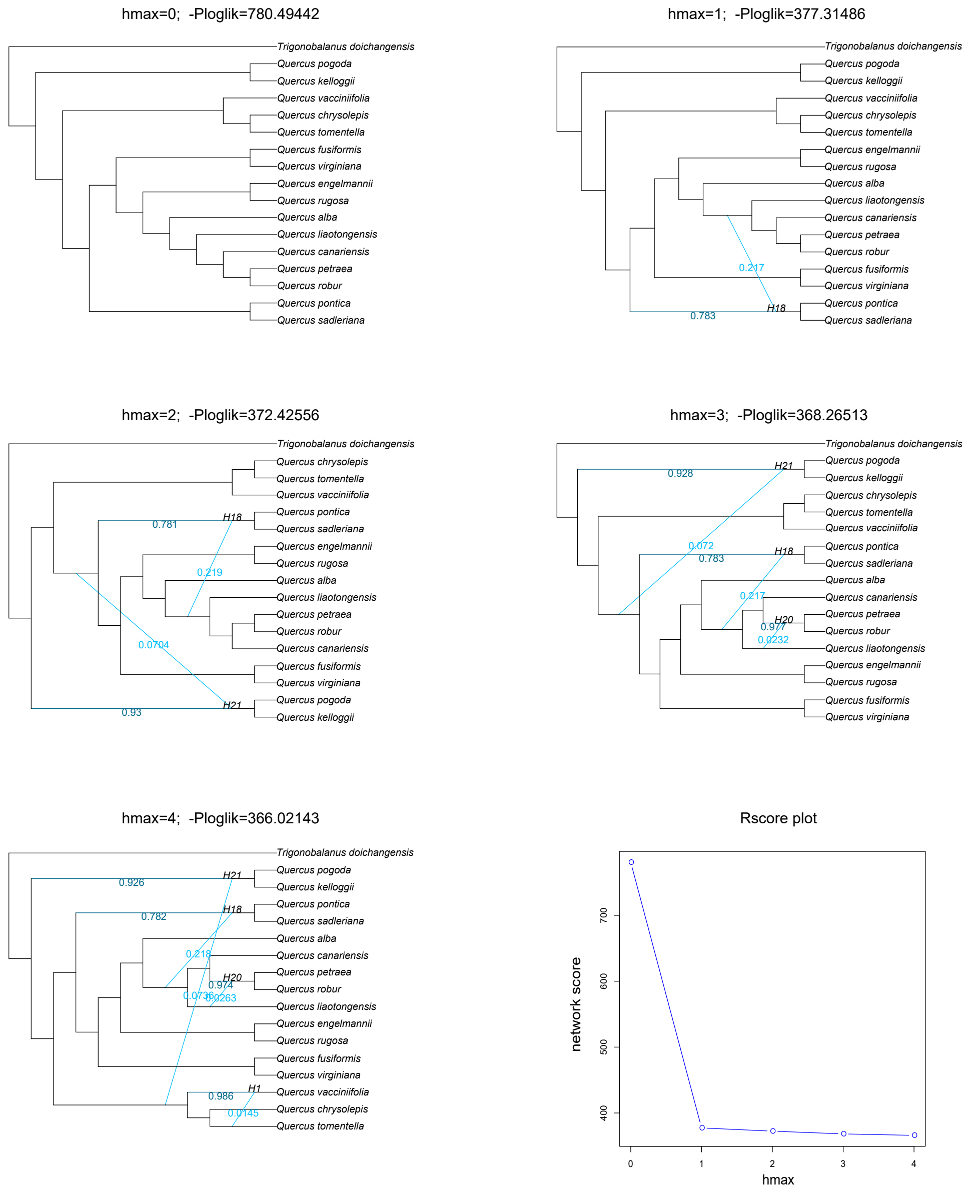

hmax=0; -Ploglik=780.49442
Trigonobalanus doichangensis
Quercus pogoda
Quercus kelloggii
Quercus vacciniifolia
Quercus chrysolepis
Quercus tomentella
Quercus fusiformis
Quercus virginiana
Quercus engelmannii
Quercus rugosa
Quercus alba
Quercus liaotongensis
Quercus canariensis
Quercus petraea
Quercus robur
Quercus pontica
Quercus sadleriana
hmax=1; -Ploglik=377.31486
0.217
H18
0.783
hmax=2; -Ploglik=372.42556
H18
0.781
0.219
0.0704
H21
0.93
hmax=3; -Ploglik=368.26513
H21
0.928
0.072
0.783
H18
0.217
H20
0.977
0.0232
hmax=4; -Ploglik=366.02143
H21
0.926
H18
0.782
0.218
H20
0.974
0.0736
0.0263
H1
0.986
0.0145
Rscore plot
network score
hmax
400
500
600
700
0
1
2
3
4

(b)

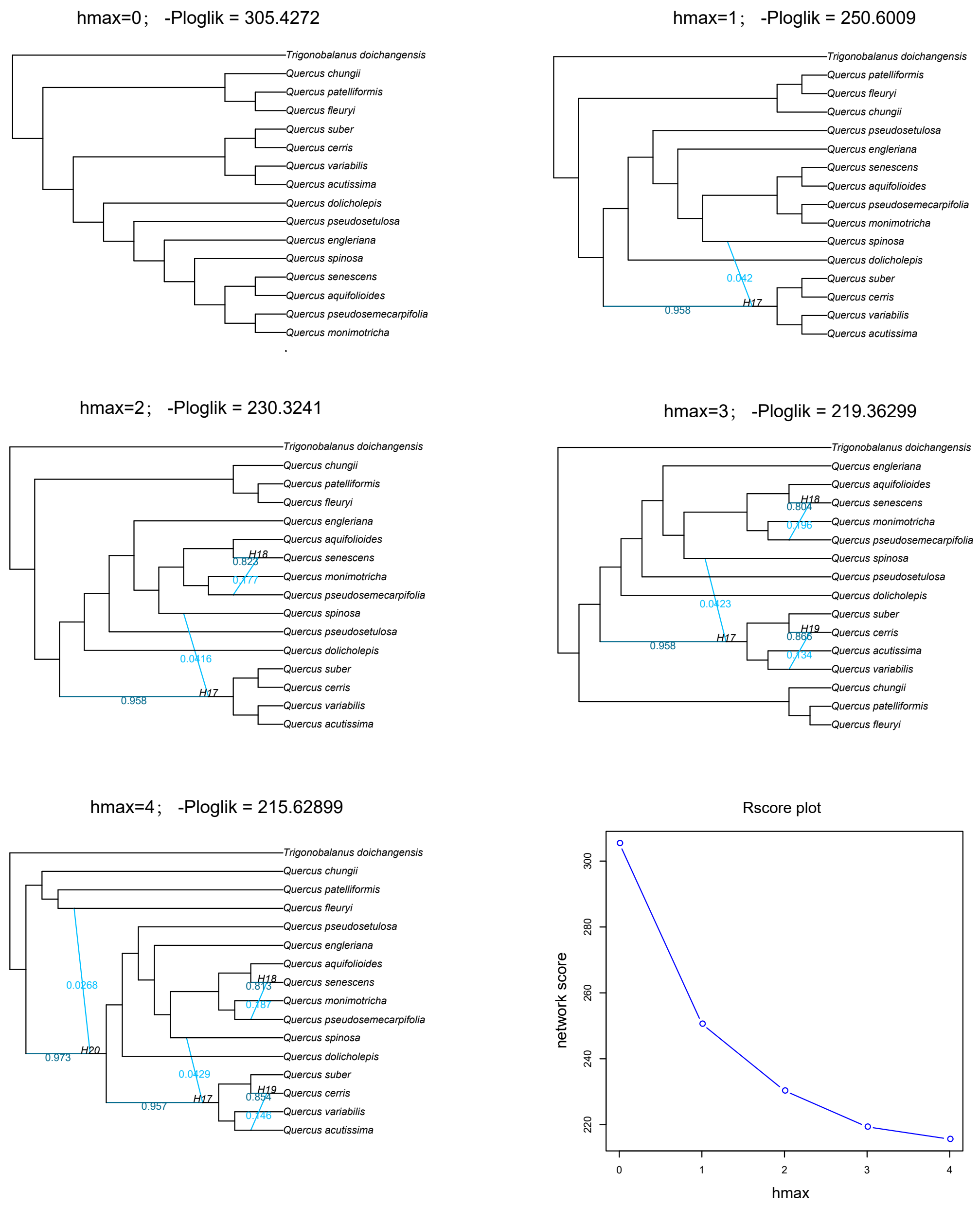

hmax=0； -Ploglik = 305.4272
Trigonobalanus doichangensis
Quercus chungii
Quercus patelliformis
Quercus fleuryi
Quercus suber
Quercus cerris
Quercus variabilis
Quercus acutissima
Quercus dolicholepis
Quercus pseudosetulosa
Quercus engleriana
Quercus spinosa
Quercus senescens
Quercus aquifolioides
Quercus pseudosemecarpifolia
Quercus monimotricha
hmax=1； -Ploglik = 250.6009
Trigonobalanus doichangensis
Quercus patelliformis
Quercus fleuryi
Quercus chungii
Quercus pseudosetulosa
Quercus engleriana
Quercus senescens
Quercus aquifolioides
Quercus pseudosemecarpifolia
Quercus monimotricha
Quercus spinosa
Quercus dolicholepis
0.042
H17
0.958
Quercus suber
Quercus cerris
Quercus variabilis
Quercus acutissima
hmax=2； -Ploglik = 230.3241
Trigonobalanus doichangensis
Quercus chungii
Quercus patelliformis
Quercus fleuryi
Quercus engleriana
Quercus aquifolioides
H18
0.823
Quercus senescens
Quercus monimotricha
0.177
Quercus pseudosemecarpifolia
Quercus spinosa
Quercus pseudosetulosa
Quercus dolicholepis
0.0416
Quercus suber
Quercus cerris
H17
0.958
Quercus variabilis
Quercus acutissima
hmax=3； -Ploglik = 219.36299
Trigonobalanus doichangensis
Quercus engleriana
Quercus aquifolioides
H18
0.804
Quercus senescens
Quercus monimotricha
0.196
Quercus pseudosemecarpifolia
Quercus spinosa
Quercus pseudosetulosa
Quercus dolicholepis
0.0423
Quercus suber
H19
0.866
Quercus cerris
0.958
H17
Quercus acutissima
0.134
Quercus variabilis
Quercus chungii
Quercus patelliformis
Quercus fleuryi
hmax=4； -Ploglik = 215.62899
Trigonobalanus doichangensis
Quercus chungii
Quercus patelliformis
Quercus fleuryi
Quercus pseudosetulosa
Quercus engleriana
Quercus aquifolioides
0.0268
H18
0.813
Quercus senescens
Quercus monimotricha
0.187
Quercus pseudosemecarpifolia
Quercus spinosa
H20
0.973
Quercus dolicholepis
0.0429
Quercus suber
H19
0.854
Quercus cerris
0.957
H17
Quercus variabilis
0.146
Quercus acutissima
Rscore plot
network score
300
280
260
240
220
0
1
2
3
4
hmax

(c)

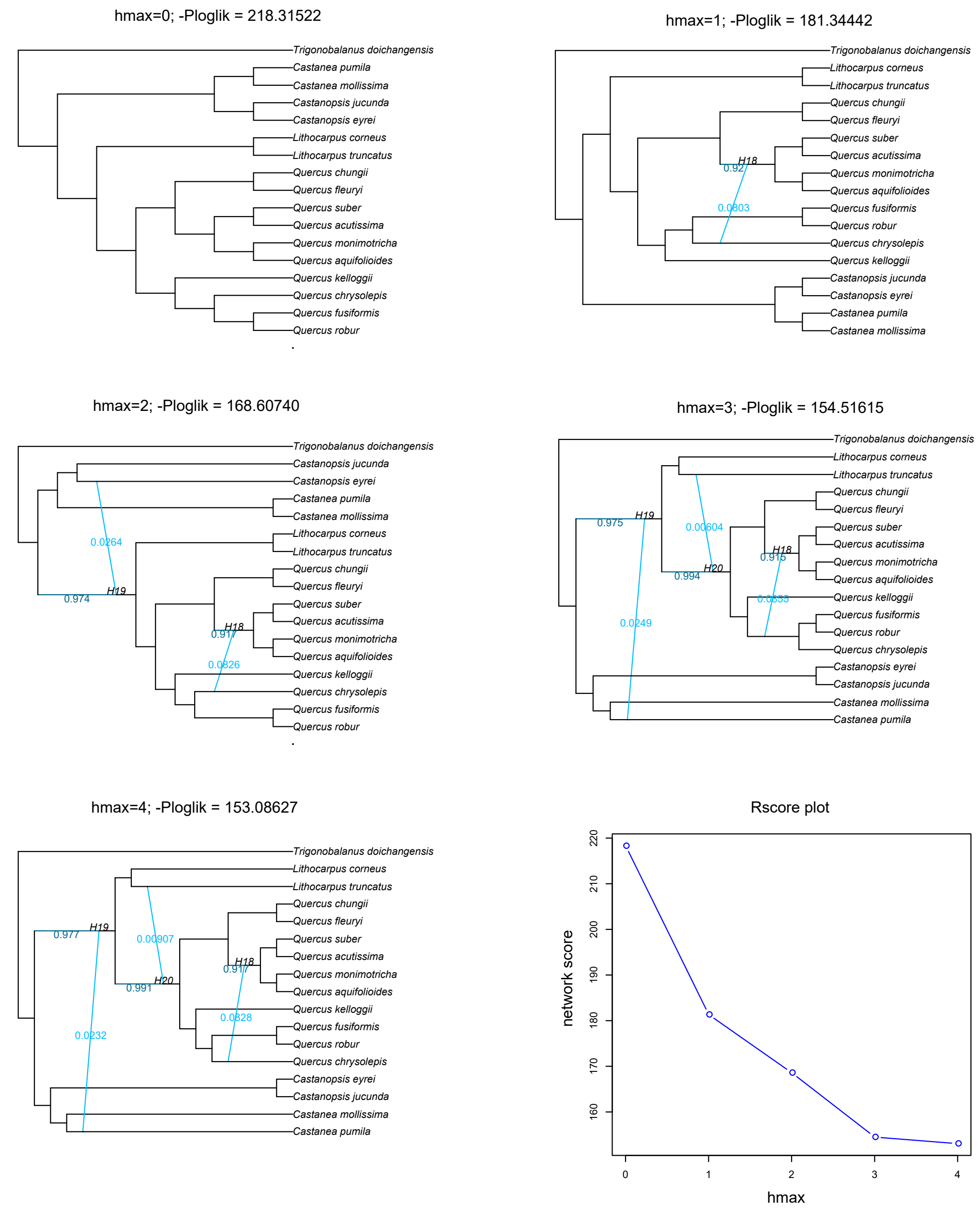
hmax=0; -Ploglik = 218.31522
Trigonobalanus doichangensis
Castanea pumila
Castanea mollissima
Castanopsis jucunda
Castanopsis eyrei
Lithocarpus corneus
Lithocarpus truncatus
Quercus chungii
Quercus fleuryi
Quercus suber
Quercus acutissima
Quercus monimotricha
Quercus aquifolioides
Quercus kelloggii
Quercus chrysolepis
Quercus fusiformis
Quercus robur
hmax=1; -Ploglik = 181.34442
Trigonobalanus doichangensis
Lithocarpus corneus
Lithocarpus truncatus
Quercus chungii
Quercus fleuryi
Quercus suber
Quercus acutissima
Quercus monimotricha
Quercus aquifolioides
Quercus fusiformis
Quercus robur
Quercus chrysolepis
Quercus kelloggii
Castanopsis jucunda
Castanopsis eyrei
Castanea pumila
Castanea mollissima
H18
0.92
0.0803
hmax=2; -Ploglik = 168.60740
Trigonobalanus doichangensis
Castanopsis jucunda
Castanopsis eyrei
Castanea pumila
Castanea mollissima
Lithocarpus corneus
Lithocarpus truncatus
Quercus chungii
Quercus fleuryi
Quercus suber
Quercus acutissima
Quercus monimotricha
Quercus aquifolioides
Quercus kelloggii
Quercus chrysolepis
Quercus fusiformis
Quercus robur
0.0264
0.974
H19
H18
0.917
0.0826
hmax=3; -Ploglik = 154.51615
Trigonobalanus doichangensis
Lithocarpus corneus
Lithocarpus truncatus
Quercus chungii
Quercus fleuryi
Quercus suber
Quercus acutissima
Quercus monimotricha
Quercus aquifolioides
Quercus kelloggii
Quercus fusiformis
Quercus robur
Quercus chrysolepis
Castanopsis eyrei
Castanopsis jucunda
Castanea mollissima
Castanea pumila
0.975
H19
0.00604
H18
0.915
H20
0.994
0.0835
0.0249
hmax=4; -Ploglik = 153.08627
Trigonobalanus doichangensis
Lithocarpus corneus
Lithocarpus truncatus
Quercus chungii
Quercus fleuryi
Quercus suber
Quercus acutissima
Quercus monimotricha
Quercus aquifolioides
Quercus kelloggii
Quercus fusiformis
Quercus robur
Quercus chrysolepis
Castanopsis eyrei
Castanopsis jucunda
Castanea mollissima
Castanea pumila
0.977
H19
0.00907
H20
0.991
H18
0.917
0.0828
0.0232
Rscore plot
network score
220
210
200
190
180
170
160
0
1
2
3
4
hmax

(d)

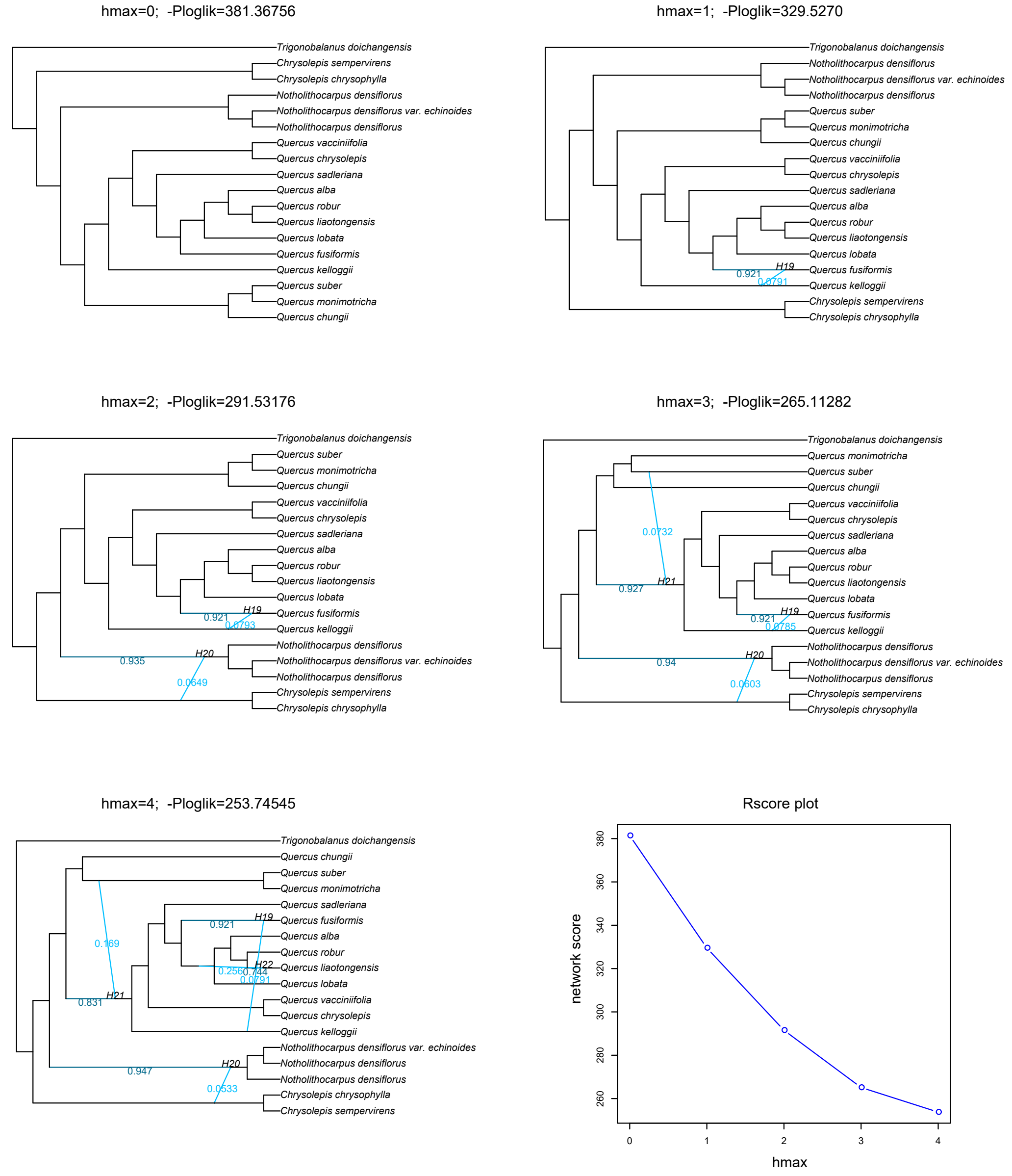

Supplementary Figure 10. Gene flow among Fagaceae lineages inferred by SNaQ analyses. (a) Within subgenus *Quercus*, (b) within subgenus *Cerris*, (c) among genera *Castanea*, *Castanopsis*, *Lithocarpus* and *Quercus*, (d) among genera *Chrysolepsis*, *Notholithocarpus* and *Quercus*. In each plot, phylogenetic networks allowing for zero (hmax = 0) to four (hmax = 4) hybridization events and network score of each hmax value were shown. The blue lines indicate hybrid edges. The light blue and dark blue numbers indicate estimated inheritance probabilities from major and minor parental species, respectively.

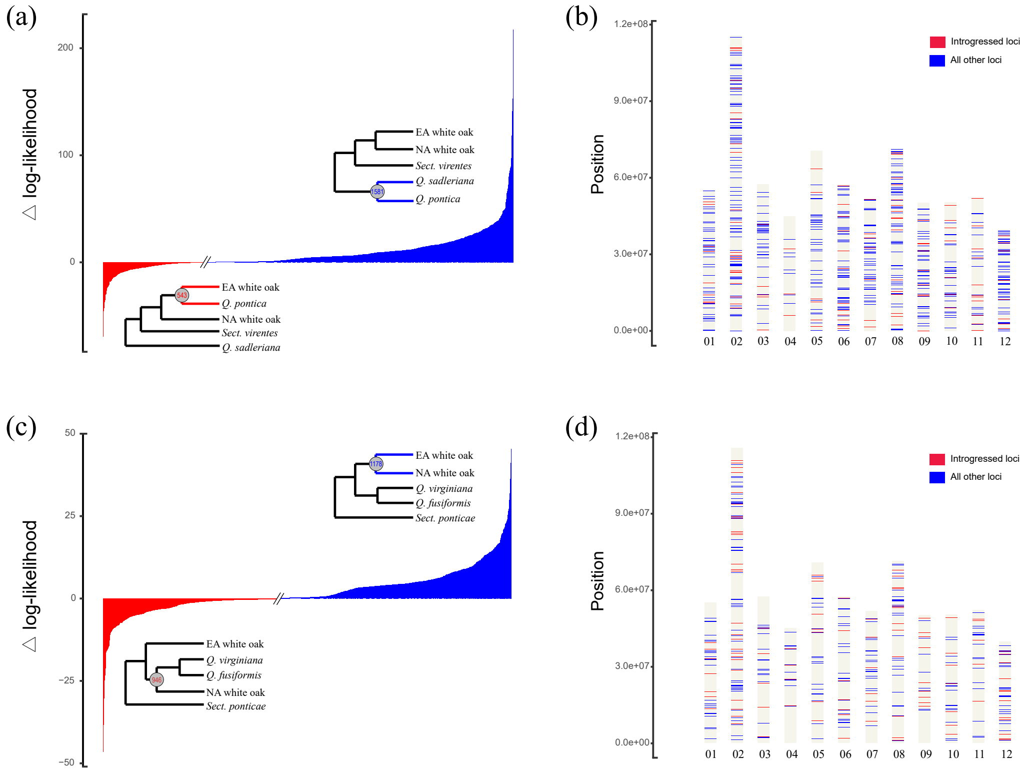

Supplementary Figure 11. Phylogenetic signals for alternative topologies. (a) Distribution of per gene Δlog-likelihood calculated between the concordant and alternative topology for the placements of *Q. pontica*. Embedded trees indicated alternative positions of *Q. pontica*, and the number on trees indicate the number of gene trees in which a given placement of *Q. pontica* was inferred. (b) Genes identified as potentially introgressed between Eurasian white oak (EA white oak) and *Q. pontica* (based on gene tree topologies in (a)) were mapped to the *Q. robur* genome and highlighted in red ($N = 543$). All other loci sampled in this study were shown in blue. (c) Distribution of per gene Δlog-likelihood calculated between the concordant and alternative topology with different placements of North American white oak (NA white oak). Embedded trees indicated alternative position of NA white oak, and the number on trees indicate the number of gene trees in which a given placement of NA white oak was inferred. (d) Genes identified as potentially introgressed between NA white oak and section *Virentes* (based on gene tree topologies in (b)) were mapped to the *Q. robur* genome and highlighted in red ($N = 946$). All other loci sampled in this study were shown in blue. In (a) and (b) Genes are ordered base on their Δlog-likelihood values, and those with Δlog-likelihood near zero (between -0.2 and 0.2) have been removed for clarity.

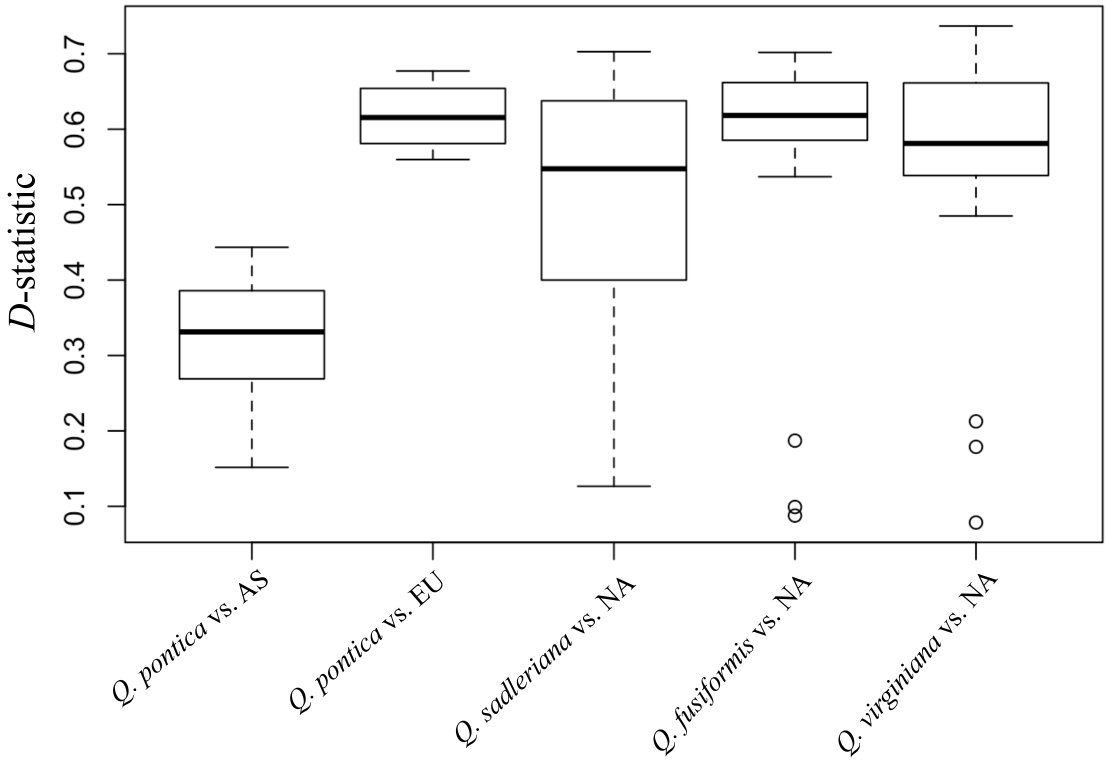

Supplementary Figure 12. Boxplot of *D*-statistics estimated in shared IBD regions for white oaks (section *Quercus*) vs. sections *Pontincae* and *Virentes*. Each box summarizes a set of *D*-statistic tests performed on trios in the format ((H1,H2),H3) with different combinations of H1 and H2 species and a fixed H3 species. Both H1 and H2 were white oaks, but represent different lineages. For example, if H2 was a North American white oak, then H1 was sampled from European or Asian white oaks. EU = European white oak; AS = Asian white oak; NA = North American white oak. For *Q. pontica* vs. AS, H1 = NA species, H2 = AS species and H3 = *Q. pontica*. For *Q. pontica* vs. EU, H1 = NA species, H2 = EU species and H3 = *Q. pontica*. For *Q. sadleriana* vs. NA, H1 = EU or AS species, H2 = NA species and H3 = *Q. sadleriana.* For *Q. fusformis* vs. NA, H1 = EU or AS species, H2 = NA species and H3 = *Q. fusformis*. For *Q. virginiana* vs. NA, H1 = EU or AS species, H2 = NA species and H3 = *Q. virginiana*. Significant gene flow (positive *D*-statistics) was detected between H2 and H3 species for all trios described above. In these box plots, the sample size n = 12 and 21 trios for *Q. pontica* vs. AS and *Q. pontica* vs. EU, respectively; n = 36 for *Q. sadleriana* vs. NA, *Q. fusiformis* vs. NA and *Q. virginiana* vs. NA,

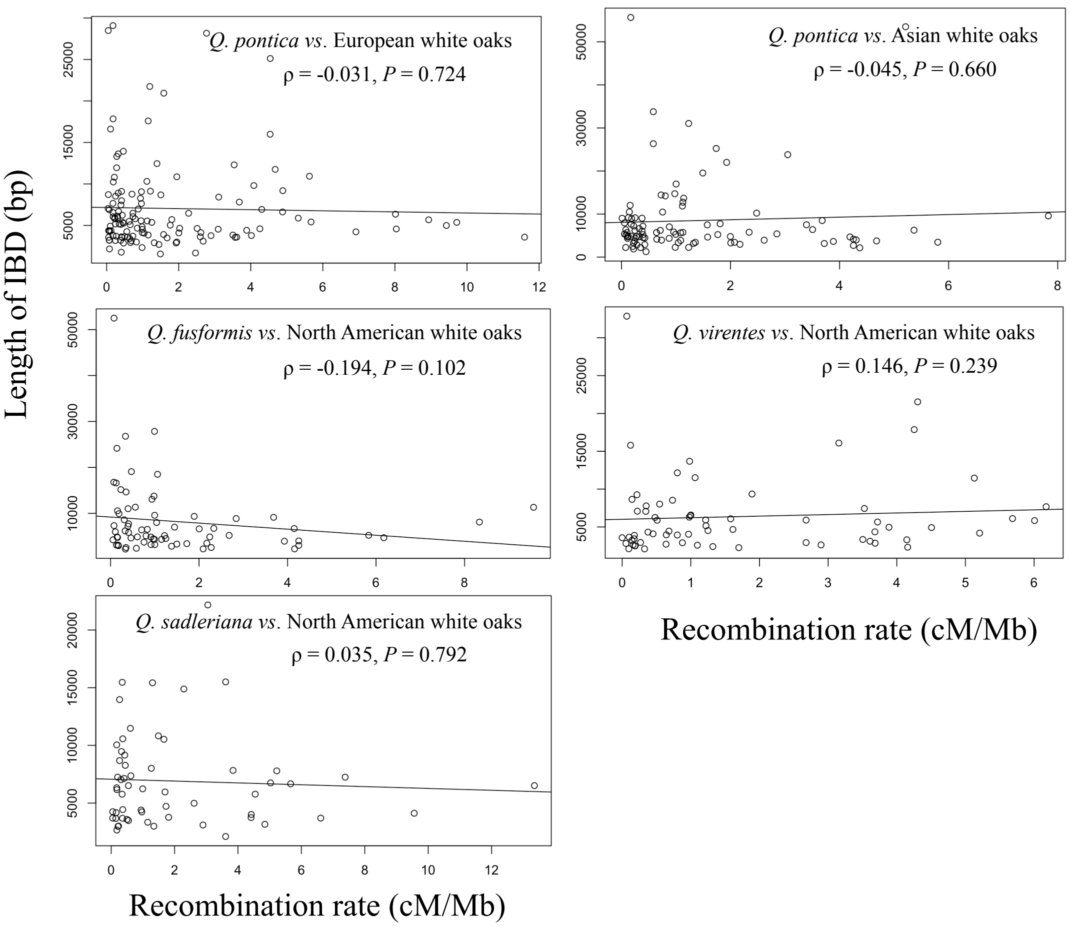

Supplementary Figure 13. Correlation between the length of identity-by-descent (IBD) blocks and recombination rate. In each plot, correlation and significance are tested with spearman's rank correlation test, two-sided.

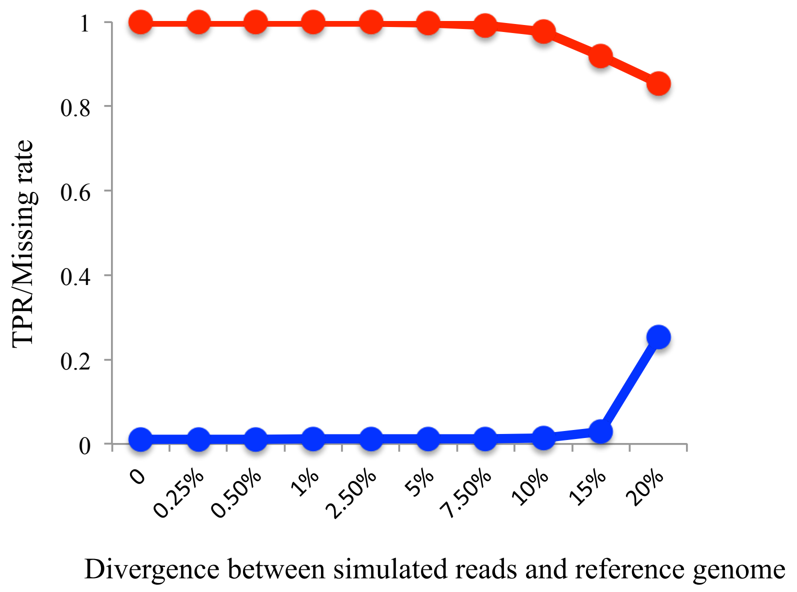

Supplementary Figure 14. The TPR (true positive rate, red) and missing rate (blue) as a function of the nucleotide divergence between target and reference genomes. TPR was defined as TP/(TP + FP), where TP is total number of called genotypes identical to real genotypes and FP is total number of called genotypes different from real genotypes.

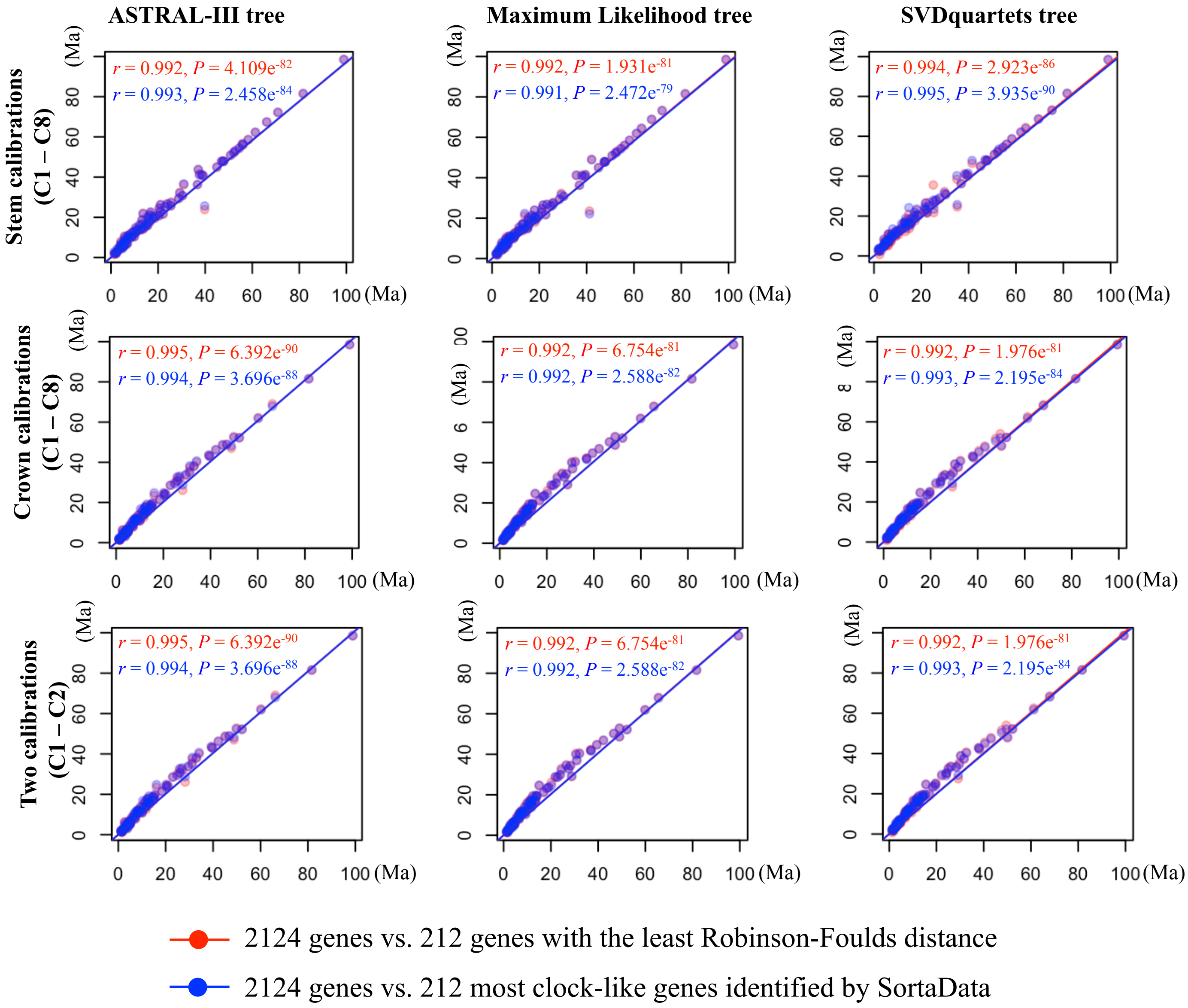

Supplementary Figure 15. Pearson's correlation coefficient (*r*) between estimated divergence time based on all 2124 genes (horizontal axis) and reduced datasets (vertical axis). Each plot shows a result of different combination of chronogram and fossil calibration scheme (See Supplementary Figure S5 for details). *P*-value is computed by Pearson's correlation test, two-sided. Ma, million years ago.

(a)

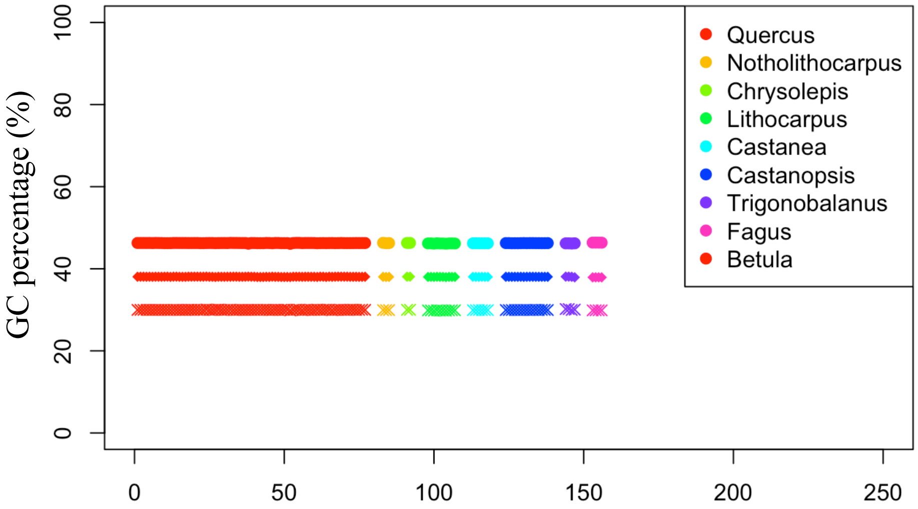

(b)

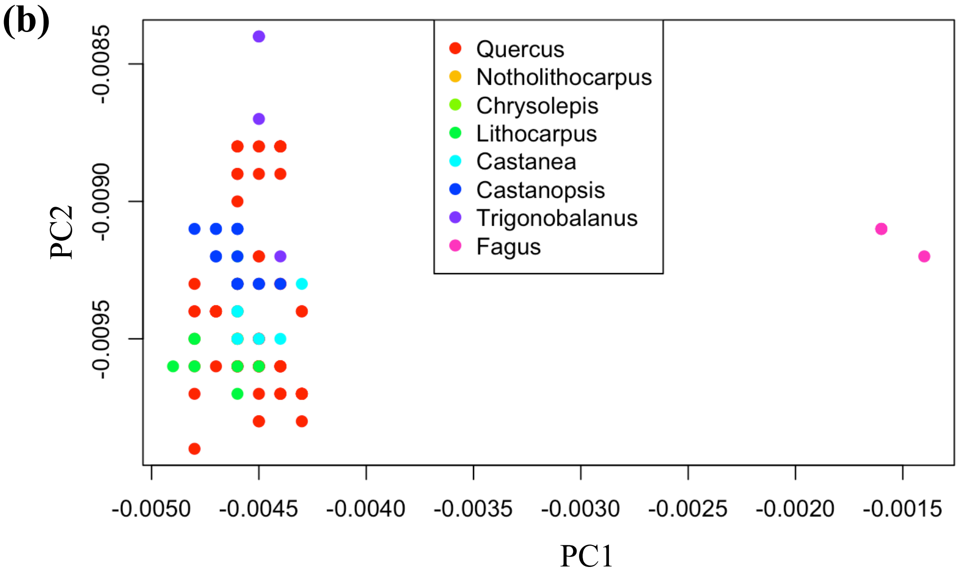

Supplementary Figure S16. (a) Nucleotide GC contents and (b) Relative synonymous codon usage (RSCU) in plastome for Fagaceae species.

Supplementary Table 1 Details of fossil calibrations for estimating divergence times.

Node	Node Label	Max Age (Ma)	Min Age (Ma)	Crown calibration node	Stem calibration node	Taxon	Collection, specimen numbers	Apomorphy-based diagnosis	Taxon ref.	Locality, stratigraphic level	Age constraint	Ref. age constraint	Remarks
Fagaceae-family	C1	82	81	Fagus_lucida|Quercus_fabri	Fagus_lucida|Quercus_fabri	Fagoideae PT 1	IPUW 7513/165	Pollen, monad, oblate to spheroidal, 14–24 µm (P axis); tricolporate, tectate, scabrate (LM), rugulate, minutely fossulate (SEM), rugulae up to 2.5 µm; multi-branched and intertwined, tips of rugulae not protruding (SEM).	Ref[1]	Elk Basin, Wyoming, USA	Biostratigraphic, magnetostratigraphic, and chronometric dating	Ref[1]	NA
Castanopsis-genus	C2	52.2	52.2	Castanopsis_eyrei|Castanea_pumila_2	Castanopsis_eyrei|Castanea_pumila_2	*Castanopsis rothwellii*	Museo Paleontológico Egidio Feruglio (MEF), Trelew, Argentina; MPEF-Pb 6433a and MPEF-Pb 6433b	Infructescence with castenoid floral remnants; cupules with maturing fruits, asymmetrically valved and sutured, solitary; fully enclosing single nuts	Ref[2]	Laguna del Hunco, Tufolitas Laguna del Hunco, La Huitrera Formation, Argentina	40Ar-40Ar ages; two paleomagnetic reversals, especially a 52.22 ± 0.22 Ma 40Ar-40Ar age on sanidine from the middle of the sequence	Ref[2]	Uncertain fossil placement on stem or crown node of Castanopsis. Denk et al.[3] reported that "it is impossible to decide whether Castanopsoidea[4] and Castanopsis rothwellii represent stem Castaneoideae/Fagaceae, are extinct sister lineages of Castanea-Castanopsis, or belong to the modern genus." Thus, we set this fossil as the stem age of Castanopsis

Quercus – genus	C3	56	56	Quercus_chungii|Quercus_fabri	Quercus|Notholithocarpus	*Quercus* type 1A	University of Vienna, Department of Palaeontology, unnumbered SEM stub	Pollen monad, prolate, 28 µm (P axis), tricolporate, microrugulate, perforate, microverrucate	Ref[5]	St. Pankraz (Salzburg, Austria); inner neritic marly claystone of the South-Helvetic thrust unit	Calcareous nannoplankton and dinoflagellates	Ref[6]	Calcareous nannoplankton: Ericsonia subpertusa, Discoaster multiradiatus; the latter is the zonal marker of calcareous nannoplankton Zone NP9 and occurs up into Zone NP11, indicating a Late Thanetian to Early Ypresian age; dinoflagellates: Apectodinium makes up 62% of the total marine palynoflora
Trigonobalanoid	C4	38	34	Trigonobalanus_verticillata|Trigonobalanus_doichangensis	Trigonobalanus_verticillata|Trigonobalanus_doichangensis	Trigonobalanoid sp 1 & sp 2	Hoffeins Amber Collection, University of Göttingen, GZG.BST.21995, GZG.BST.21996	Staminate inflorescences with high similarity to modern Trigonobalanus verticillata	Ref[7]	Baltic Amber, Late Eocene	Lithological and biostratigraphic studies, including pollen, spores and phytoplankton	Ref[7]	According to Crepet & Nixon[4] "fossilized fruit of Trigonobalanus succinea[8], also from Baltic amber (Kalininigrad), has the diagnostic combination of triangular cross section and capitate-discoid stigmas that characterizes the modern species of Trigonobalanus." This specimen is lost, but a drawing by Conwentz[9] is similar to the modern genus.

<table>
<tr><td>section Lobatae</td><td>C5</td><td>47.87</td><td>47.87</td><td>Quercus_agrifolia|Quercus_pagoda</td><td>Quercus_agrifolia|Quercus_fabri</td><td>Quercus PT 1 aff. Group Lobatae</td><td>University of Vienna, Department of Palaeontology, unnumbered SEM stub</td><td>Pollen, monad, prolate, tricolporate, pori small, nexine thinner or as thick as sexine, scabrate (LM), tectum microverrucate, fossulate, perforate (SEM), microechinate suprasculpture; identical tectum sculpture is found in modern species of sect. Lobatae</td><td>Ref[1]</td><td>Princeton Chert (British Columbia, Canada); uppermost part of Allenby Fm, Ashnola Shale, chert bed 43</td><td>Radiometric (^{40}Ar–^{39}Ar and Pb–U)</td><td>Ref[10]</td><td>48.7 Ma from ash layer #22 from the Ashnola shale</td></tr>
<tr><td>section Quercus</td><td>C6</td><td>45</td><td>45</td><td>Quercus_virginiana|Quercus_fabri</td><td>Quercus_pontica|Quercus_fabri</td><td>Quercus L.</td><td>US188-5894; US188-4580, 188-5731, 188-5893, 188-5894, 188-5903, 188-6150, 188-6151, 188-6152, 188-8943 to 8948, 188-8949, 188-8950, 188-8951, 188-8952, 188-8953, 188-8954, 188-8955, 188-8956, 188-8957, 188-8958, 188-8959, 188-8960, 188-8961, 188-8962, 188-8963, 188-8964, 188-8965, 188-8966, 505-5970- to 5977, 505-6147 to 6149; leaf material stored at University of Saskatchewan</td><td>Leaves, lobed; leaf shape and lobes identical to a clade of deciduous white oaks comprising Quercus aliena forma acuteserrata[11]; white oak pollen[12]</td><td>Refs[11,12]</td><td>"Stream site", localities US 188, 505, Geodetic Hills, Axel Heiberg Island; Buchanan Lake Formation</td><td>Brontothere remains</td><td>Ref[13]</td><td>Interpreted to exclude sections Ponticae and Virentes.</td></tr>
<tr><td>section Ilex</td><td>C7</td><td>47.8</td><td>37.8</td><td>Quercus_guyavaefolia|Quercus_dolicholepis</td><td>Quercus_guyavaefolia|Quercus_dolicholepis</td><td>Quercus type 1B</td><td>University of Vienna, Department of Palaeontology, unnumbered SEM stub</td><td>Pollen, monad, prolate, tricolporate, tectum microrugulate (rod-like)</td><td>Ref[5]</td><td>Changchang flora, Hainan Island, South China; Changchang Formation</td><td>Palynology, fish, turtles, crocodiles, mammals; magnetostratigraphy</td><td>Ref[5]</td><td>NA</td></tr>
</table>

section *Cerris*	C8	34	30	Quercus_castaneifolia|Quercus_chenii2	Quercus_castaneifolia|Quercus_chenii2	*Quercus gracilis*	Specimens stored at FEGI; holotype Spec. 96/9182 [FEGI]; other specimens 9182/118, 256, 271, 339, 491, 585, 624, 804, 857, 900, 1013	Leaves, dentate, leaf dentitions typically with bristle-like extensions, secondary veins densely spaced, straight, parallel, evenly spaced; leaves identical to East Asian members of sect. *Cerris*	Ref[14]	Kraskino, South Primorye, Russian Far East; Fatashinskaya Suite	Correlation with adjacent radiometrically dated sites[15,16]	Refs[15,16]	NA

NA, not applicable
Ma, million years ago

Supplementary Table 2 Total number (upper triangular) and total length (lower triangular) of shared identity-by-descent (IBD) between sections of *Quercus*. Numbers on indicate diagonal line number/length of shared IBDs within sections. The length of IBD was in unit of kb.

	AS	EU	NA	Sect. Virentes	*Quercus pontica*	*Quercus sadleriana*	Sect. *Protobalanus*	Sect. *Lobatae*	Sect. *Ilex*	Sect. *Cerris*	Sect. *Cyclobalanopsis*
AS	143-6630 /1.3-106.6	8-60	1-4	0-1	9-25	1-5	0-2	0	0	0-1	0
EU	0.067-0.667	1442-2505 /15.2-27.7	1-17	0-3	55-66	5-7	0-1	0	0	0	0
NA	0-0.044	0.005-0.127	97-10813 /0.7-134.6	15-34	3-7	13-20	0-2	0	0	0	0
Sect. Virentes	0-0.007	0-0.014	0.11-0.306	9166/72.8	1	3-4	0	0			0
Q. pontica	0.065-0.204	0.353-0.519	0.02-0.05	0.004	NA	2102	1-3	0	0	0	0
Q. sadleriana	0-0.032	0.022-0.062	0.079-0.223	0.016-0.021	13.078	NA	8-22	0	0	0	0
Sect. *Protobalanus*	0-0.025	0-0.015	0-0.037	0	0-0.011	0.051-0.145	4962-6540 /37.7-53.5	0	0	0	0
Sect. *Lobatae*	0	0	0	0	0	0		15-9410 /0.1- 94.7	0	0-4	0
Sect. *Ilex*	0	0	0	0	0	0	0	0	1-9194 /0-93.1		0-3
Sect. *Cerris*	0-0.003	0	0	0	0	0	0	0	0-0.034	1-11200 /0-113.1	0
Sect. *Cyclobalanopsis*	0	0	0	0	0	0	0	0	0-0.025	0	1-4478/0-34.7

NA = North American white oak; EU = European white oak; AS = Asian white oak.

NA, not applicable due to small sample size (n = 1).

Supplementary Table 3 Comparison of recombination rate (cM/Mb, mean ± standard error) between IBD blocks and genomic background. *P*-values were estimated by Mann-Whitney *U*-test, two-sided.

Lineage-pairs	IBD blocks	Genomic background	*W*	*P*-value
Quercus fusiformis vs. North American white oaks (*N* = 72)	1.486 ± 0.222	1.587 ± 0.044	80866.0	0.705
Quercus virginiana vs. North American white oaks (*N* = 67)	1.737 ± 0.261	1.587 ± 0.044	68734.5	0.381
Quercus sadleriana vs. North American white oaks (*N* = 58)	2.037 ± 0.345	1.587 ± 0.044	60146.5	0.494
Quercus pontica vs. Aisan white oaks (*N* = 96)	1.419 ± 0.162	1.587 ± 0.044	107838.5	0.662
Quercus pontica vs. European white oaks (*N* = 131)	1.786 ± 0.196	1.587 ± 0.044	139432.5	0.596

IBD, identity-by-descent

Supplementary Table 4 Gene Ontology (GO) categories overrepresented for genes located in identity-by-descent (IBD) blocks.

GO term	Ontology	Description	False discovery rate (FDR)*
***Quercus sadleriana vs*. North American white oaks**			
GO:0080016	MF	E-beta-caryophyllene synthase activity	0.00237
GO:0080017	MF	alpha-humulene synthase activity	0.00237
GO:0010334	MF	sesquiterpene synthase activity	0.00373
GO:0010333	MF	terpene synthase activity	0.00499
GO:0016838	MF	carbon-oxygen lyase activity, acting on phosphates	0.00499
GO:0051761	BP	sesquiterpene metabolic process	0.00568
GO:0051762	BP	sesquiterpene biosynthetic process	0.00568
***Quercus virginiana vs*. North American white oaks**			
GO:0000974	CC	Prp19 complex	0.00030
GO:0071014	CC	post-mRNA release spliceosomal complex	0.00044
GO:0000393	BP	spliceosomal conformational changes to generate catalytic conformation	0.00063
GO:0005684	CC	U2-type spliceosomal complex	0.00108
GO:0071013	CC	catalytic step 2 spliceosome	0.00115
GO:0005681	CC	spliceosomal complex	0.00838

*We applied Fisher's exact test to estimate the significance of enrichment, and corrected multiple testing by Benjamini–Hochberg FDR

MP, molecular function; BP, biological process; CC, cellular component.

Note, No GO categories were overrepresented for genes located in IBD blocks of the three other lineage-pairs:
Quercus pontica vs. European and Aisan white oaks, *Quercus fusiformis vs*. North American white oaks.

Supplementary Table 5 Comparision of called genotypes from two different reference genome (*Quercus robur* and *Castanea mollissima*) for speceis of five Fagaceae genera.

	Number of sites with called genotypes		Missing rate (%)		
Sample ID	*Q. robur*	*C. mollissima*	*Q. robur*	*C. mollissima*	TPR (%)
Trigonobalanus doichangensis_1	1710467	1811170	9.53	4.20	95.73
Trigonobalanus doichangensis_2	1706881	1809902	9.72	4.27	95.62
Trigonobalanus verticillata	1710499	1812596	9.53	4.13	95.64
Trigonobalanus excelsa	1707831	1810125	9.73	4.29	95.61
Castanopsis carlesii_1	1713384	1815605	9.38	3.97	95.75
Castanopsis carlesii_2	1713382	1815424	9.38	3.98	95.76
Castanopsis eyrei	1713573	1815457	9.37	3.98	95.76
Castanopsis fabri	1713960	1815610	9.35	3.97	95.78
Castanopsis fargesii	1714158	1815660	9.34	3.97	95.79
Castanopsis fissa	1713848	1815543	9.35	3.97	95.78
Castanopsis hainanensis	1713379	1815538	9.38	3.97	95.75
Castanopsis hystrix	1713838	1815807	9.35	3.96	95.75
Castanopsis jucunda_1	1713888	1815467	9.35	3.98	95.78
Castanopsis jucunda_2	1713812	1815599	9.35	3.97	95.76
Castanopsis lamontii	1712200	1814862	9.44	4.01	95.72
Castanopsis nigrescens	1714083	1815180	9.34	3.99	95.83
Castanopsis orthacantha	1713557	1815615	9.37	3.97	95.76
Castanopsis sclerophylla_1	1714375	1815666	9.32	3.97	95.80
Castanopsis sclerophylla_2	1714011	1815504	9.34	3.97	95.78
Chrysolepis chyrsophylla	1715068	1815703	9.29	3.96	95.82
Chrysolepis sempervirens	1714407	1815661	9.32	3.97	95.79
Lithocarpus uvariifolius	1714932	1814922	9.29	4.01	95.83
Lithocarpus amygdalifolius	1713464	1814404	9.37	4.03	95.77
Lithocarpus calophyllus	1714258	1814690	9.33	4.02	95.80
Lithocarpus corneus	1714720	1814741	9.31	4.02	95.84
Lithocarpus haipinii	1713014	1814675	9.40	4.02	95.75
Lithocarpus hancei	1713727	1814872	9.36	4.01	95.76
Lithocarpus longanoides	1714150	1814860	9.34	4.01	95.79
Lithocarpus longipedicellatus	1714477	1814710	9.32	4.02	95.83
Lithocarpus longipedicellatus	1714340	1814836	9.33	4.01	95.81
Lithocarpus truncatus	1714582	1814902	9.31	4.01	95.80
Notholithocarpus densiflorus_1	1714505	1815390	9.32	3.98	95.80
Notholithocarpus densiflorus_2	1714343	1815018	9.33	4.00	95.81
Notholithocarpus densiflorus var echinoides	1714750	1814723	9.30	4.02	95.84

TPR, ratio of identical genotypes called from both *Q. robur* and *C. mollissima.*

Supplementary Table 6 The Nucleotide divergence (d_{XY}) between species on 2124 nuclear orthologous genes.

Species	*Fagus sylvatica*	*Castanea mollissima*	*Quercus robur*	*Quercus lobata*	*Quercus suber*
Fagus sylvatica	0				
Castanea mollissima	7.69%	0			
Quercus robur	7.46%	2.26%	0		
Quercus lobata	7.50%	2.31%	0.65%	0	
Quercus suber	7.58%	2.43%	1.67%	1.70%	0

Supplementary Table 7 Alignment length and polymorphic sites of the 76 plastid genes in 122 Fagaceae species and one outgroup.

Gene name	Alignment length	No. of polymorphic site	Gene name	Alignment length	No. of polymorphic site
psbA	1068	79	*petG*	114	10
matK	1515	323	*psaJ*	135	25
rps16	270	112	*rpl33*	222	39
psbK	186	24	*rps18*	360	87
psbI	111	9	*rpl20*	354	48
atpA	1524	137	*rps12*	375	10
atpF	555	69	*clpP*	591	63
atpH	246	14	*psbB*	1527	121
atpI	771	93	*psbT*	108	8
rps2	711	58	*psbN*	132	11
rpoC2	4143	599	*psbH*	222	17
rpoC1	2067	620	*petB*	648	58
rpoB	3231	1494	*petD*	510	73
petN	90	7	*rpoA*	996	131
psbM	105	4	*rps11*	417	46
psbD	1062	832	*rpl36*	114	11
psbC	1464	880	*infA*	147	64
psbZ	189	16	*rps8*	405	50
rps14	303	29	*rpl14*	369	31
psaB	2205	134	*rpl16*	408	40
psaA	2253	117	*rps3*	666	76
ycf3	507	27	*rps19*	294	53
rps4	609	53	*rpl2*	864	59
ndhJ	477	39	*rpl23*	282	11
ndhC	363	34	*ycf2*	6900	312
atpE	402	44	*ndhB*	1539	30
atpB	1497	136	*rps7*	468	10
rbcL	1449	118	*ndhF*	1443	205
accD	1551	298	*rpl32*	126	15
psaI	114	22	*ccsA*	957	140
ycf4	555	66	*psaC*	246	23
cemA	690	80	*ndhE*	306	22
petA	966	112	*ndhG*	531	84
psbJ	123	7	*ndhI*	498	55
psbL	117	8	*ndhA*	1101	152
psbF	120	9	*ndhH*	1182	136
psbE	252	10	*rps15*	282	64
petL	96	12	*ycf1*	6018	1943

Supplementary Table 8 The number of species recorded in each genus of Fagaceae and each section of the genus *Quercus*.

Taxon	No. of species	Sampled species in this study
Genus *Fagus*	10	2
Genus *Trigonobalanus*	3	3
Genus *Castanea*	12	5
Genus *Castanopsis*	120	12
Genus *Lithocarpus*	334	10
Genus *Notholithocarpus*	2	2
Genus *Chrysolepis*	2	2
Genus *Quercus*	423	54
Section *Lobatae*	124	7
Section *Quercus*	146	15
Section *Virentes*	7	2
Section *Ponticae*	2	2
Section *Protobalanus*	5	3
Subgenus *Cerris*	139	25
Section *Cerris*	13	6
Section *Ilex*	36	10

Supplementary Table 9 Substitution saturation on the 1st + 2nd codon, and the 3rd codon.

No. of OTUs	Iss.obs	Iss.cSym	*P*-value (Iss.obs vs. Iss.cSym)	Iss.cAsym	*P*-value (Iss.obs vs. Iss.cAsym)
1st and 2nd codon					
4	0.038	0.859	0	0.853	0
8	0.045	0.854	0	0.778	0
16	0.053	0.845	0	0.699	0
32	0.061	0.818	0	0.596	0
3rd codon					
4	0.055	0.861	0	0.849	0
8	0.06	0.847	0	0.764	0
16	0.065	0.853	0	0.68	0
32	0.075	0.82	0	0.58	0

OUT, operational taxonomic unit.

Iss.obs, index of substitution saturation for subsets of OTUs randmomly sampled from full dataset.

Iss.cSym, the predicted Iss value under symmetrical tree.

Iss.cAsym, the predicted Iss value under asymmetrical tree.

P-value is computed by Chi-square (x^2) test, two-sided.

References

1 Grímsson, F., Grimm, G. W., Zetter, R. & Denk, T. Cretaceous and Paleogene Fagaceae from North America and Greenland: Evidence for a Late Cretaceous split between Fagus and the remaining Fagaceae. *Acta Palaeobotanica* **56**, 247-305 (2016).

2 Wilf, P., Nixon, K. C., Gandolfo, M. A. & Cuneo, N. R. Eocene Fagaceae from Patagonia and Gondwanan legacy in Asian rainforests. *Science* **364**, eaaw5139 (2019).

3 Denk, T. *et al.* Comment on "Eocene Fagaceae from Patagonia and Gondwanan legacy in Asian rainforests". *Science* **366**, 814-814 (2019).

4 Crepet, W. L. & Nixon, K. C. Earliest megafossil evidence of Fagaceae: Phylogenetic and biogeographic implications. *Am. J. Bot.* **76**, 842-855 (1989).

5 Hofmann, C. C. in *8th European Palaeobotany-Palynology Conference* 119 (Hungarian Natural History Museum, Budapest, 2010).

6 Hofmann, C. C., Mohamed, O. & Egger, H. A new terrestrial palynoflora from the Palaeocene/Eocene boundary in the northwestern Tethyan realm (St. Pankraz, Austria). *Rev. Palaeobot. Palynol.* **166**, 295-310 (2011).

7 Sadowski, E. M., Schmidt, A. R. & Denk, T. Staminate inflorescences with *in situ* pollen from Eocene Baltic amber reveal high diversity in Fagaceae (oak family). *Willdenowia* **50**, 405-517 (2020).

8 Forman, L. L. Trigonobalanus a new genus of Fagaceae with notes on the classification of the family. *Kew Bull* **17**, 381–396 (1964).

9 Conwentz, H. in *Die Flora des Bernsteins und ihre Beziehungen zur Flora der Tertiärformation und der Gegenwart 2* (eds H. R. Göppert & A. Menge) (Engelmann, 1886).

10 Greenwood, D. R., Pigg, K. B., Basinger, J. F. & DeVore, M. L. A review of paleobotanical studies of the Early Eocene Okanagan (Okanogan) Highlands floras of British Columbia, Canada, and Washington, USA. *Canadian Journal of Earth Sciences* **53**, 548-564 (2016).

11 McIver, E. E. & Basinger, J. F. Early Tertiary floral evolution in the Canadian high arctic. *Ann. Mo. Bot. Gard.* **86**, 523-545 (1999).

12 McIntyre, D. J. Pollen and spore flora of an Eocene forest, eastern Axel Heiberg Island, N.W.T. . *Geological Survey of Canada Bulletin*, 83-98 (1991).

13 Eberle, J. J. & Greenwood, D. R. Life at the top of the greenhouse Eocene world-A review of the Eocene flora and vertebrate fauna from Canada's High Arctic. *Geological Society of America Bulletin* **124**, 3-23 (2012).

14 Pavlyutkin, B. I. The genus *Quercus* (Fagaceae) in the Early Oligocene Flora of Kraskino, Primorskii Region. *Paleontol. J.* **49**, 668-676 (2015).

15 Akhmetiev, M., Walther, H. & Kvacek, Z. Mid-latitude Palaeogene floras of Eurasia bound to volcanic settings and palaeoclimatic events – experience obtained from the Far East of Russia (Sikhote-Alin') and Central Europe (Bohemian Massif). *Acta Musei Nationalis Pragae, Series B: Historia Naturalis* **65**, 61-129 (2009).

16 Tanai, T. & Uemura, K. Lobed oak leaves from the tertiary of East Asia with reference to the oak phytogeography of the northern hemisphere. *Transactions and Proceedings of the Palaeontological Society of Japan New Series* **173**, 343-365 (1994).
