# Supplementary material for: Phylogenomic analyses highlight innovation and introgression in the continental radiations of Fagaceae across the Northern Hemisphere
Source: Nat Commun. 2022 Mar 14;13:1320. doi: 10.1038/s41467-022-28917-1 (PMC8921187; doi:10.1038/s41467-022-28917-1)
Supplement: Supplementary file 3 — Description of Additional Supplementary Files [file 41467_2022_28917_MOESM3_ESM.pdf]

Description of Additional Supplementary Files

File Name: Supplementary Data 1
Description: List of species sampled.

File Name: Supplementary Data 2
Description: Alignment length and polymorphic sites of the 2124 orthologous genes in 122 Fagaceae individulas and one outgroup.

File Name: Supplementary Data 3
Description: The 236 trios with significant gene flow detected by *D*-statistic tests. The *D*-statistic tests were performed on trios in the format ((H1, H2), H3), and positive *D* values indicate gene flow between H2 and H3. The significance of *D* values was tested by a two-sided standard block-jackknife procedure implemented in Dsuite with default parameters, and adjusted by Bonferroni correction for multiple comparisons.

File Name: Supplementary Data 4
Description: Six Lists of genes with the best information for datating. Each list of genes were identified a combiantion of "gene-shopping" method and reference topology.
