# Supplementary material for: Phylogenomic analyses highlight innovation and introgression in the continental radiations of Fagaceae across the Northern Hemisphere
Source: Nat Commun. 2022 Mar 14;13:1320. doi: 10.1038/s41467-022-28917-1 (PMC8921187; doi:10.1038/s41467-022-28917-1)
Supplement: Supplementary file 8 — Reporting Summary [file 41467_2022_28917_MOESM8_ESM.pdf]

nature portfolio

Corresponding author(s): Paul S. Manos & Baosheng Wang

Last updated by author(s): Feb 5, 2022

Reporting Summary

Nature Portfolio wishes to improve the reproducibility of the work that we publish. This form provides structure for consistency and transparency in reporting. For further information on Nature Portfolio policies, see our Editorial Policies and the Editorial Policy Checklist.

Statistics

For all statistical analyses, confirm that the following items are present in the figure legend, table legend, main text, or Methods section.

n/a	Confirmed	
☐	☒	The exact sample size (*n*) for each experimental group/condition, given as a discrete number and unit of measurement
☐	☒	A statement on whether measurements were taken from distinct samples or whether the same sample was measured repeatedly
☐	☒	The statistical test(s) used AND whether they are one- or two-sided<br>*Only common tests should be described solely by name; describe more complex techniques in the Methods section.*
☒	☐	A description of all covariates tested
☐	☒	A description of any assumptions or corrections, such as tests of normality and adjustment for multiple comparisons
☐	☒	A full description of the statistical parameters including central tendency (e.g. means) or other basic estimates (e.g. regression coefficient) AND variation (e.g. standard deviation) or associated estimates of uncertainty (e.g. confidence intervals)
☐	☒	For null hypothesis testing, the test statistic (e.g. *F*, *t*, *r*) with confidence intervals, effect sizes, degrees of freedom and *P* value noted<br>*Give P values as exact values whenever suitable.*
☐	☒	For Bayesian analysis, information on the choice of priors and Markov chain Monte Carlo settings
☐	☒	For hierarchical and complex designs, identification of the appropriate level for tests and full reporting of outcomes
☐	☒	Estimates of effect sizes (e.g. Cohen's *d*, Pearson's *r*), indicating how they were calculated

Our web collection on statistics for biologists contains articles on many of the points above.

Software and code

Policy information about availability of computer code

Data collection	No software was used to collect the data.
Data analysis	OrthoFinder v2.3.12 was used to obtain orthologous genes; BLAST+ v2.10 was used to identify homologous sequences; Trimmomatic v0.39, BWA v0.7.17 and GATK v4.2 were used to filtered reads and called SNPs; lastZ/Multiz pipeline was used to perform genome alignment; WGSM was used to simulate short reads; NOVOPlasty v4.2 was used to assemble plastomes; PAG v1.0 and Geneious v7.1.4 were used to annotate the plastomes; MAFFT 7.221 and Bioedit v.7.2 was used to aligned sequences; RAxML v8.2.12, MrBayes v3.2.6, ASTRAL-III v5.7.3, SVDquartets v1.0 and PAUP v4.0a152 were used to constructed phylogenetic trees; PartitionFinder2 v1.1 was used to determine the partitioning strategy and model; MCMCTree v4.9j in the PAML v4.9j was used to calculated divergence time; Baseml v4.9j in PAML v4.9j was used to estimate substitution rates a strict molecular clock; Tracer v1.7 and LogCombiner v1.10 were used to confirm the convergence across each run; SortaData was used to identify most clock-like loci; BAMMtools v2.5.0 was used to estimate the diversification rate; BAMMtools v2.1.5 was used to summarize and plot samples generated by BAMM analyses; PHYPARTS was used to assess topological concordance; DAMBE v7.035 and GCUA were used to evaluate substitutional saturation and codon-usage bias; DENDROPY v.4.1.0 was used to perform coalescent simulation; Phylonet v2.4 was used to count the number of extra lineages in observed and simulated trees; Dsuite v0.3 and SNaQ implemented in the package PhyloNetworks v0.12.0 were used to assessed gene flow; Beagle v4.1 was used to identify shared haplotypes; R package topGO v2.43.0 was use to performed Gene Ontology (GO) analyses.<br>Codes used in this study have been deposited in the Dryad digital data repository (https://doi.org/10.5061/dryad.vq83bk3tc).

For manuscripts utilizing custom algorithms or software that are central to the research but not yet described in published literature, software must be made available to editors and reviewers. We strongly encourage code deposition in a community repository (e.g. GitHub). See the Nature Portfolio guidelines for submitting code & software for further information.

Data

Policy information about availability of data

All manuscripts must include a data availability statement. This statement should provide the following information, where applicable:

- Accession codes, unique identifiers, or web links for publicly available datasets
- A description of any restrictions on data availability
- For clinical datasets or third party data, please ensure that the statement adheres to our policy

Short reads of whole genome sequencing data generated in this study have been deposited in Genbank under accession code PRJNA773751 (https://www.ncbi.nlm.nih.gov/sra/?term=PRJNA773751). Alignments of nuclear genes and plastomes generated in this study have been deposited in the Dryad digital data repository (https://doi.org/10.5061/dryad.vq83bk3tc). Previously published genome assemblies are available in Genbank under accession numbers PRJEB14544, PRJEB24056, PRJNA527178, PRJNA433227, PRJEB19898, MG386401 and NC036929. Source data are provided with this paper.

Field-specific reporting

Please select the one below that is the best fit for your research. If you are not sure, read the appropriate sections before making your selection.

☐ Life sciences ☐ Behavioural & social sciences ☒ Ecological, evolutionary & environmental sciences

For a reference copy of the document with all sections, see nature.com/documents/nr-reporting-summary-flat.pdf

Ecological, evolutionary & environmental sciences study design

All studies must disclose on these points even when the disclosure is negative.

Study description	We investigate the timing and pattern of major macroevolutionary events and ancient genome-wide signatures of hybridization across Fagaceae. We re-sequenced whole genomes of fagaceae species, called SNPs from 2,124 nuclear loci, and assembled full plastomes. With these data, we conducted phylogenomic analyses to characterize the diversification of Fagaceae and identify admixed genomes due to ancient gene flow within the first complete family-wide phylogenetic context.
Research sample	We sampled 122 individuals from 91 species of Fagaceae. Details of samples are presented in Supplementary Data 1.
Sampling strategy	Samples were collected either from natural populations or botanical garden. We chose species that are taxonomically well-understood and easily identified. We sampled 122 individuals from 91 species representing all eight currently recognized genera of Fagaceae and all eight recognized sections of genus Quercus. The sample size is enough to answer the question focused on this study, i.e. the timing and pattern of major macroevolutionary events and ancient genome-wide signatures of hybridization across Fagaceae.
Data collection	Fresh leaves were collected from living trees in natural population or cultivated plants grown from wild-collected seed, and then dried with silica gel (by Alejandra Jaramillo, Biao-Feng Zhou, David Hillis, Dylan Burge, Jason Love, Jie Gao, Jim Costa, Ming Qing, Shuai Yuan, Yong-Jie Guo and Yong Shi). Total genomic DNA was extracted from silica-dried leaf tissue (by Qing-Qing An, Xue-Yan Chen and Yi-Ye Liang), and sequenced on the illumina NovaSeq platform at Novogene (Beijing, China).
Timing and spatial scale	We sampled species from July 2018 to September 2019, during the tree growing seasons when the fresh leaves are available. We conducted worldwide sampling from Asian, Europe, North America and South America (Supplementary Data 1).
Data exclusions	We excluded short reads and SNPs with low quality using standard procedure of genomic study to obtain high quality data.
Reproducibility	All analyses were rerun by three times and generated identical results, suggesting that our results are reproducible.
Randomization	n/a - we performed phylogenomic analyses where nothing to be randomized.
Blinding	n/a - we performed phylogenomic analyses where blinding was not relevant.

Did the study involve field work? ☒ Yes ☐ No

Field work, collection and transport

Field conditions	Samples were collected from deciduous forest or tropical-subtropical evergreen forest. The local environment in field is not relevant to the study question.
Location	We sampled species from multiple countries: China, Colombia, Sweden, UK and USA. Details of the sample sites are listed in Supplementary Data 1.
Access & import/export	None of the plant materials was collected from nature protection area, and no collection permission was required.
Disturbance	We only collected 3-5 leaves per tree, which would not disturb the habitats.

Reporting for specific materials, systems and methods

We require information from authors about some types of materials, experimental systems and methods used in many studies. Here, indicate whether each material, system or method listed is relevant to your study. If you are not sure if a list item applies to your research, read the appropriate section before selecting a response.

Materials & experimental systems

n/a	Involved in the study
☒	☐ Antibodies
☒	☐ Eukaryotic cell lines
☒	☐ Palaeontology and archaeology
☒	☐ Animals and other organisms
☒	☐ Human research participants
☒	☐ Clinical data
☒	☐ Dual use research of concern

Methods

n/a	Involved in the study
☒	☐ ChIP-seq
☒	☐ Flow cytometry
☒	☐ MRI-based neuroimaging
